# Genomic analysis of multi-drug resistant coagulase-negative staphylococci from healthy humans and animals revealed unusual mechanisms of resistance and CRISPR-Cas system

**DOI:** 10.1007/s10123-024-00577-9

**Published:** 2024-09-17

**Authors:** Idris Nasir Abdullahi, Carmen Lozano, Javier Latorre-Fernández, Myriam Zarazaga, Marc Stegger, Carmen Torres

**Affiliations:** 1 Area of Biochemistry and Molecular Biology, OneHealth-UR Research Group, University of La Rioja, Logroño, Spain; 2 Department of Medical Laboratory Science, Faculty of Allied Health Sciences, College of Medical Sciences, Ahmadu Bello University, Zaria, Nigeria; 3 Department of Bacteria, Parasites, and Fungi, Statens Serum Institut, Copenhagen, Denmark; 4 Antimicrobial Resistance and Infectious Diseases Laboratory, Harry Butler Institute, Murdoch University, Murdoch, WA Australia

**Keywords:** Staphylococci, *cfr*, Linezolid resistance, CRISPR-Cas systems, Plasmids, Enterotoxins

## Abstract

**Background:**

Coagulase-negative staphylococci (CoNS) are evolving as major reservoirs and vectors of unusual and critical antimicrobial resistance (AMR) mechanisms.

**Materials and methods:**

In this study, the genomic characterization of 26 multidrug-resistant (MDR)-CoNS (*S. borealis*, *S. saprophyticus*, *S. sciuri*, *S. hominis*, *S. epidermidis*, *S. pasteuri*, *S. hyicus*, *S. simulans*, *S. haemolyticus*, and *S. arlettae*) previously obtained from the nasal cavity of healthy nestling storks, humans who had no contact with animals, pigs, and pig farmers, as well as dogs and dog owners from Spain was performed. High-quality draft genomes obtained by Illumina sequencing technology were used to determine their resistome, virulome, mobile genetic elements, and CRISPR-Cas types.

The relatedness of three CoNS species with publicly available genomes was assessed by core-genome single nucleotide polymorphisms (SNPs).

**Results:**

AMR genes to all classes of antibiotics in staphylococci were detected including unusual ones (*mecC*, *ermT*, and *cfr*), of which their corresponding genetic organizations were analyzed. About 96.1% of the MDR-CoNS strains harbored diverse adherence or immune evasion genes. Remarkably, one enterotoxin-C and -L-carrying *S. epidermidis*-ST595 strain from a nestling stork was detected. Moreover, various plasmid bound-biocide resistance genes (*qacACGJ*) were identified in 34.6% of the MDR-CoNS. Two genes that encode for cadmium and zinc resistance (*cadD*, *czrC*) were found, of which *czrC* predominated (42.3%). Complete CRISPR-Cas system was detected in 19.2% of the CoNS strains, of which *cas*-1, -2, and -9 predominated, especially in 75% of the *S. borealis* strains. The phylogenetic analysis identified clusters of related *S. epidermidis* lineages with those of other countries (SNP < 100). Also, highly related *S. borealis* isolates (SNP < 10) from pigs was confirmed for the first time in Spain.

**Conclusion:**

These findings showed that various ecological niches harbor CoNS that presented MDR phenotypes mediated by multiple AMR genes carried by mobile genetic elements with relatively low frequency of intact CRISPR-Cas systems. Furthermore, the transmission of some CoNS species in humans and animals is strongly suggested.

**Supplementary Information:**

The online version contains supplementary material available at 10.1007/s10123-024-00577-9.

## Introduction

The members of the *Staphylococcus* genus are part of the normal microbiota of the nose and skin of humans and animals (including many avian species) (Szczuka et al. 2023). In addition, coagulase-positive staphylococci could occasionally cause clinical diseases mediated by highly potent virulence genes (Aqel et al. 2023). However, not every staphylococcal virulence gene is expressed. Instead, the expression of the genes is usually restricted to times and places and regulated by bacterial factors (Grazul et al. 2023). Over the last years, some coagulase-negative staphylococci (CoNS) species (as *S. epidermidis*, *S. haemolyticus*, or *S. hominis*) emerged as a cause of opportunistic infections such as those in septicaemic children, or in patients with immunosuppression or medical implants, among others (França et al. 2021; Heilmann et al. 2019). Most of other CoNS species are unfrequently implicated in human or animal infections, being often highly susceptible to antimicrobial agents (Merrild et al. 2023; Santoiemma et al. 2020; Argemi et al. 2019). However, there have been sporadic reports of some *S. pasteuri* causing endocarditis, whereas *S. hyicus*, *S. chromogenes*, *S. lentus*, and *S. sciuri* are considered etiological agents of exudative epidermitis with zoonotic potentials (Kirk et al. 2022; Kalai et al. 2021; Li et al. 2021). Moreover, *S. saprophyticus* contracted from contaminated food have long been implicated in urinary tract infections in young teenagers (Lawal et al. 2021a, b). Much more recently, whole-genome sequence data of CoNS species have led to the identification and characterization of numerous putative virulence factors (Argemi et al. 2019). Furthermore, CoNS could acquire clinically relevant and critical antimicrobial resistance (AMR) genes and transmit them across other species and hosts through various mobile genetic elements (mobilome) (Rossi et al. 2020). Specifically, *S. haemolyticus* has been ranked as the most antibiotic-resistant species among the CoNS (Kranjec et al. 2021). The transferability of AMR genes between different *Staphylococcus* species has been strongly suggested by the sequence similarity of their associated mobilome, especially plasmids (Souza-Silva et al. 2022).

The *mecA* gene, its staphylococcal cassette chromosome (SCC*mec*) carrying element, and the arginine catabolic mobile element (ACME) originated from CoNS were acquired by *S. aureus* (Shokrollahi et al. 2022). *mecC*-carrying CoNS have also been reported from many countries but in very low frequencies (Loncaric et al. 2019). Previously thought to be a wildlife MRSA trait, the continuous spread of the SCC*mec*-bound *mecC* gene in CoNS highlights their potential role in the evolutionary origin and genetic transfer to MRSA (Abdullahi et al. 2023a).

Most methicillin-resistant CoNS (MRCoNS) strains are often found to be resistant to other non-betalactam antibiotics except for glycopeptides, which have long been utilized in the treatment of staphylococcal infections (Chajęcka-Wierzchowska et al. 2023). As the AMR epidemic keeps expanding, the few methicillin-resistant staphylococcal infections that are treated using oxazolidinones (Gostev et al. 2021) could have promoted the emergence, spread, and persistence of linezolid resistance, as some mechanisms mediated by ARGs (*cfr*, *poxtA*, and *optrA*) are carried by plasmids (Bai et al. 2019; Dortet et al. 2018). However, high-level linezolid resistance could be caused by non-transferable mechanisms mediated by mutations in the 23S rDNA, and in the ribosomal proteins L3, L4, and L22 (Ruiz-Ripa et al. 2021).

The clustered regularly interspaced short palindromic repeats (CRISPR) and CRISPR-associated proteins (Cas) are RNA-based adaptive immunity to protect and are utilized by many bacteria against invading mobile genetic elements (MGEs) (Tao et al. 2022a). Hence, the CRISPR–Cas system might be a potential means to prevent the acquisition of plasmid and phage invasion and even horizontal transfer of AMR genes in staphylococci (Murugesan and Varughese 2022). There are two categories of CRISPR-Cas, which are based on their proteins’ structures, constituents, and modes of action (Nishimasu and Nureki 2017). The Class 1 CRISPR-Cas uses multiple protein effector complexes to break down nucleotides and can be subdivided into types -I, -III, and -IV, whereas the Class 2 CRISPR-Cas utilizes single-protein effector complexes to break down nucleotides, of which it is subdivided into types -II, -V, and -VI (Shmakov et al. 2015; Makarova et al. 2015). The types II-CRISPR-Cas systems have largely been studied and have successfully been used to delete antimicrobial resistance genes (ARGs) due to their relatively simple structures (Tao et al. 2022a). Moreover, the Type I CRISPR-Cas systems have been developed and manipulated to eliminate ARGs (Tao et al. 2022a). In this regard, certain CRISPR-Cas system prevents foreign nucleotides (such as plasmids and phages) from evading the bacteria thereby limiting the acquisition of ARGs (Tao et al. 2022a).

The genetic characterization of CoNS is necessary to understand their evolution and source distribution, reservoir hosts, and vectors of AMR transmission. In this regard, certain animal hosts such as the pigs and human workers in pig farm environments are believed to be under high antibiotic pressure and carry staphylococci presenting a high-level multidrug resistance (MDR) phenotype. However, animals in the wildlife may be at low antibiotic pressure as they are rarely exposed to antimicrobial agents (Abdullahi et al. 2021). It is worth mentioning that the ecology and epidemiology of AMR in CoNS could be different from that of *S. aureus* because the CoNS species could present different and diverse AMR profiles*.* In this study, the genomic characterization of 26 multidrug resistant-CoNS (resistant to ≥ four classes of antimicrobial agents) previously obtained from the nasal cavity of healthy humans without animal contact, nestling storks, pigs and pig owners, as well as dogs and their owners from Spain were performed by Illumina technology.

## Materials and methods

### Coagulase-negative staphylococci strains in this study

A total of 516 non-repetitive CoNS strains were obtained in previous studies (Abdullahi et al. 2023a; b; c; 2024a) from nasal samples of healthy animals and healthy humans with different types of animal contact: (a) healthy nestling storks (NS) (268 isolates); (b) healthy pigs (H-P) and pig farmers (H-PF) (75 isolates); (c) healthy dogs (H–D) and dog owners (H-DO) (130 isolates); and (d) healthy humans who had no contact with animals (HH^−^) (113 isolates). The antimicrobial susceptibility of these isolates was previously determined by disk diffusion tests, and the presence of ARGs by PCR (Abdullahi et al. 2023a; b; c; 2024a). From this collection, 26 CoNS isolates of 10 species (*S. borealis*, *S. saprophyticus*, *S. sciuri*, *S. hominis*, *S. epidermidis*, *S. pasteuri*, *S. hyicus*, *S. simulans*, *S. haemolyticus*, and *S. arlette*) were selected to be further characterized in the present study by whole genome sequencing (WGS), and they were of the following origins: NS (*n* = 4); H-P and H-PF (*n* = 14); H–D and H-DO (*n* = 4); and HH^−^ (*n* = 4). The selection criteria of the strains included were as follows: (i) CoNS that presented an MDR phenotype for four or more classes of antibiotics, selecting one species each per host carrying this resistance phenotype; and (ii) MDR-CoNS isolates with similar AMR genes detected from humans and animals in the same ecological niche to detect potential transmission events.

The study protocols in which these isolates were recovered were reviewed and approved by the ethical research committees of the University of Zaragoza, the University of La Rioja and the University of Castilla La Mancha (Spain).

### Genome sequencing, assembly, and phylogenetic analyses

Whole genome sequencing of the selected 26 CoNS isolates was carried out on the Illumina NextSeq platform. The MagNA Pure 96 DNA Multi-Sample Kit (Life Technologies, Carlsbad, CA, USA, 4413021) was used to extract genomic DNA according to instructions provided by the manufacturers. The Qubit 1X dsDNA HS Assay Kit (Thermo Fisher Scientific, Scoresby, VIC, Australia) was used for DNA quantification, while Sequencing libraries were prepared using the Illumina Nextera XT DNA Library Preparation Kit (Illumina, San Diego, CA, USA, FC-131–1096) and sequenced on the NextSeq 500 platform (Illumina, San Diego, CA, USA) using a 300-cycle kit to obtained paired-end 150 bp reads, as previously described (Abdullahi et al. 2023d).

All the genomes analyzed in this study were de novo assembled using SPAdes (v.3.15.5), performing the in silico typing with the settings of a minimum of 90% coverage and 80% identity. First, core-genome single nucleotide polymorphisms (SNPs) between the eight *S. epidermidis* strains in this study were detected with the NASP pipeline v.1.0.0 (Sahl et al. 2016) after they were mapped together with a reference strain ATCC 14990 (GenBank accession number: GCA_006094375) and 31 previously published *S. epidermidis* genomes from different countries with similar genetic lineages from the PubMLST database (https://pubmlst.org/bigsdb?db=pubmlst_sepidermidis_strains&page=query&genomes=1) (identification [id] numbers: 32110, 32113, 32116, 41749, 42109, 43340, 43421, 43426, 43427, 43436, 43455, 43466, 43518, 43568, 43636, 43643, 43656, 43697, 43720, 43770, 43771, 43774, 43786, 43800, 43816, 43823, 43921, 44294, 44298, 44496, 44521) to obtain an *S. epidermidis* phylogenetic trees. GATK (v.4.2.2) was used to call SNPs and excluded positions featuring < 90% unambiguous variant calls and < 10 depth. IQ-TREE (v.2.1.2) was used to construct the phylogenetic trees using ModelFinder with 100 bootstraps. The graphical data was added to the phylogenies with iTOL v.6.6 (Letunic and Bork 2021). To determine the relatedness of the *S. saprophyticus* from a pig and pig farmer, we used a web-based CSI phylogeny database to obtain the SNPs by mapping the genomes to a reference *S. saprophyticus* ATCC 15305 (GenBank accession no. AP008934.1) with the default parameter, except for the minimum distance between SNPs which was disabled. Also, the SNPs of the *S. borealis* from four pigs were determined by comparing them with 16 additional publicly available genomes of *S. borealis* strains available from NCBI (GenBank accession numbers: GCA_030362885, GCA_030362875, GCA_003580835, GCA_003580835, GCA_034103225, GCA_024580895, GCA_030501495, GCA_035788295, GCA_035791815, GCA_035791575, GCA_013345165, GCA_009735325, GCA_013345185, GCA_013345175, GCA_013345205, GCA_013345195) mapped with a reference strain 7067_4#69 (GenBank accession number: GCA_001224225.1) by using the web-based CSI phylogeny database following settings similar to the ones used for *S. saprophyticus.*

### Annotation, typing, and in silico analysis of the CoNS genomes

The sequence types (STs) were determined with MLST v.2.16 (Jolley et al. 2018). Virulence factors, plasmid replicons, and antimicrobial resistance genes were identified using ABRicate v.0.9.0 and the respective databases VFDB, Plasmidfinder, and Resfinder databases from the Center for Genomic Epidemiology. Mutations associated with AMR were identified using ResFinder v4.1 (Bortolaia et al. 2020) and PointFinder (Zankari et al. 2017). Biocide and heavy metal resistance genes were identified using BACMET (Pal et al. 2014). Phaster was used to identify all prophage elements (Arndt et al. 2016). The SCC*mec* types were assigned using SCC*mec*Finder 1.2 (https://cge.food.dtu.dk/services/SCCmecFinder/). The genetic environment of the *ermT*, *cfr*, and *mecC* genes was illustrated in comparison with the reference strains using the EasyFig software.

### Determination of the CRISPR-Cas system of coagulase-negative staphylococci

The CrisprCasFinder (https://crisprcas.i2bc.paris-saclay.fr/) was used to identify the numbers of CRISPR, Cas proteins, and spacers of all the MDR-CoNS (Couvin et al. 2018). Specifically, the size of the flanking region and other parameters were set to default values. Moreover, three CoNS strains that contained larger sequences than CrisprCasFinder could handle were analyzed by the CRISPRCasMeta (https://crisprcas.i2bc.paris-saclay.fr/CrisprCasMeta/Index) applying all the default settings.

### Genome availability

All the raw genome reads generated from this study have been deposited at the European Nucleotide Archive under Study Accession no. PRJNA1023081.

### Statistical analysis

Data generated from this study reported frequencies and were presented in tables. Univariate logistic regression was to compute the odd ratio (OR) at a 95% confidence interval (95%CI) between the presence of MDR-CoNS genomes, and various mobilome with the ecological niches. Significant association at *p* < 0.05 was considered.

## Results and discussion

CoNS have long been considered reservoirs of ARGs; however, very few genomic studies have elucidated the influence of different ecological niches on the levels of ARGs and their MGEs. Moreover, there is a paucity of phylogenomic data on the transmission pathways of CoNS species and their ARGs between humans and animals.

### Resistome, mobilome, and relatedness of the 26 CoNS analyzed in this study

The phenotypes of resistance of the 26 CoNS isolates characterized in this study are shown in Supplementary Table S1, and their resistome, virulome, genetic lineages, and mobile genetic elements are represented in Table 1. As identified, all the isolates presented an MDR phenotype to 4 to 9 classes of antimicrobial agents. In this regard, the CoNS isolates with the least were those from nestling storks and with the highest those from pigs and pig farmers (Table 1). The mechanisms of resistance to most of the antibiotics were mediated by combinations of multiple antibiotic resistance genes (ARGs).

**Table 1:** Antimicrobial resistome, and metal and biocide resistance determinants in the 26 MDR-CoNS isolates and their associated mobile genetic elements

Strain ID	Species	Source/ID	ST/CC	SCC*mec*	Resistome (plasmid replicons)	No. of antibiotic classes with resistance	Metal/biocide resistance (plasmid replicons)	Other plasmid replicons	Chromosomal point mutations	Transposon (AMR genes)	IS (AMR genes)
X4922	*S. borealis*	H-P/A-8	NT	Vc	*blaZ*, *mecA*, *ermC* (*repUS12*), *lnuB*, *lsaE*, *vgaA*(*LC*), *tet*(L) (*repUS12*), *tet*(M), *tet*(45), *dfrK* (*repUS12*), *ant4′* (*repUS12*), *ant6′*, *bleO* (*repUS12*), *fexA*	7	*czrC*	*rep5b*, *rep13*, *rep19b*, *rep39*	GyrA (E84G)	Tn*554* (*fexA*)	ISSep3 (none)
X5417	*S. borealis*	H-P/B-4	NT	Vc	*mecA*, *ermA*, *ermC* (*repUS12*, *rep24c*), *vga*(*E*), *tet*(K), *tet*(L) (*repUS12*, *rep24c*), *tet*(45), *dfrK* (*repUS12*, *rep24c*), *ant4′* (*repUS12*, *rep24c*), *ant6′*, *ant9′*, *aph3′*, *bleO* (*repUS12*, *rep24c*)	6	*qacG* (*rep21*), *czrC*	None	GyrA (E84G)	None	IS256 (none),ISSep3 (none)
X5418	*S. borealis*	H-P/B-5	NT	Vc	*mecA*, *ermA*, *ermC* (*repUS12*), *vga*(*E*), *dfrK* (*repUS12*), *tet*(K), *tet*(L) (*repUS12*), *ant4′ (repUS12)*, *ant6′*, *ant9′*, *aph3′*, *bleO* (*repUS12*)	6	*qacG* (*rep21*), *czrC*	*repUS24c*	GyrA (E84G)	None	IS256, ISSep3, ISSha1
X5409	*S. borealis*	H-P*/*B-4	NT	Vc	*blaZ*, *mecA*, *ermT* (*repUS18*), *vga*(*A*)*LC*, *tet*(L), *tet*(M), *dfrK*, *aac2′-aph2″*, *ant4′*, *ant6′*, *ant9′* (*repUS18*), *aph3′*, *bleO*, *fexA*, *sat4*	8	*qacJ*, *smr*, *czrC*	*rep5e*, *rep15*, *rep19b*, *rep20*, *rep24c*, *rep39*, *repUS76*	GyrA (E84G)	Tn*558* (*fexA*)	ISsep3, ISSha1
X4944	*S. saprophyticus*	H-P/A-P10	NT	Vc	*mecA*, *ermC*, *lsaB* (*rep15*), *tet*(L) (*rep22*), *tet*(M), *tet*(45), *dfrK* (*rep22*), *ant4′* (*rep22*), *str* (*rep7a*), *fexA*, *cfr* (*rep15*)*, fusD*, (*rep10*)	7	*qacJ* (*rep21*), *czrC*	*rep19c*, *rep20*, *rep21*	None	Tn*554* (*fexA*)	ISSau9 (*cfr*, *lsaB*)
X5435	*S. saprophyticus*	H-P*/*B-P6	NT	IV (2B)	*blaZ*, *mecA*, *ermC*, *lnuB*, *lsaE*, *tet*(K) (*rep7a*), *tet*(M), *dfrC*, *dfrG*, *aac6′-aph2″*, *ant4′* (*rep22*), *ant6′*, *aph3′*, *fusD*	6	*qacJ* (*rep21*), *czrC*	*rep19c*, *rep20*, *rep21*, *rep24c*	None	None	IS256
X5462	*S. saprophyticus*	H-PF*/*B-F1	NT	IV (2B)	*blaZ*, *mecA*, *ermC* (*rep10*), *lnuB*, *lsaE*, *vga*(*A*)*V*, *tet*(K) (*rep7a*), *tet*(M), *dfrC*, *dfrG*, *aac6′-aph2″*, *ant4′* (*rep22*), *ant6′*, *aph3′*, *str*, *fusD*	6	*qacJ* (*rep21*), *czrC*	*rep20*, *rep24c*	None	None	IS256
X5776	*S. haemolyticus*	H-PF/D-F2	ST30	Vc	*blaZ*, *mecA*, *vga*(*A*)*LC* (*rep5b*), *tet*(K) (*rep7a*), *dfrG*, *aac6′-aph2″*	6	*czrC*	*rep39*, *repUS70*	GyrA (S84L)	None	None
X7059	*S. haemolyticus*	HH/34	ST30	Vc	*blaZ*, *mecA*, *msrA*, *mphC*, *tet*(K) (*rep7a*), *dfrG*, *aac6′-aph2″*, *ant4′* (*repUS12*), *bleO* (*repUS12*)	7	*czrC*	None	GyrA (S84L)	None	IS256
*X3784	*S. haemolyticus*	NS/546	ST68	V	*blaZ*, *mecA*, *lnuA* (*rep22*), *tet*(K) (*rep7a*), *dfrG*, *aac6′-aph2″*, *ant4′* (*rep22*)	5	*qacJ*, *smr*, *czrC*	*rep20*, *repUS22*	None	None	IS256
X4892	*S. sciuri*	H-P/A-P2	ST212	VIII	*mecA*, *mecA1*, *ermA* (*repUS18*), *ermC* (*rep10*), *ermB* (*repUS76*, *rep16*), *erm45*, *lnuA*, *salA*, *tet*(M) (*repUS43*), *tet*(L) (*repUS12*), *tet*(45), *dfrD* (*rep22*), *aac6′-aph2″*, *ant4′* (*repUS12*), *ant9′* (*repUS18*), *bleO* (*repUS12*), *str* (*rep7a*, *repUS18*), *fexA*	6	*qacG* (*rep21*), *czrC*	*rep19a*	None	Tn*558* (*fexA*, *salA*) Tn*6006* (none)	None
X5485	*S. epidermidis*	H-PF/B-F1	ST16/CC5	IV (2B)	*blaZ*, *mecA*, *lsaB*, *vga*(*A*)*LC* (*rep5b*), *tet*(K) (*rep7a*), *tet*(L) (*rep22*), *tet*(45), *dfrK* (*rep22*), *ant4′* (*rep22*), *str (rep7a)*, *fexA*, *cfr*, *fosB*	9	None	None	GyrA (S80F), GyrL (E84G)	None	ISSau9 (*cfr*, *lsaB*), ISSau4 (none)
X6590	*S. epidermidis*	HH/19	ST89/CC2	None	*blaZ*, *ermC* (*repUS12*), *fosB*, *mupA*	4	None	*rep7a*	None	None	None
X6628a	*S. epidermidis*	HH/22	ST210	None	*blaZ*, *lnuA*, *dfrC*, *dfrG*, *tet*(K) (*rep7a*), *ant4′* (*rep22*), *fosB*	6	None	*rep13*, *rep20*, *repUS22*	GyrA (S80F)	None	ISSau4
X9066	*S. epidermidis*	HH/46	ST173	V	*blaZ*, *mecA*, *vgaA (rep5)*, *lnuA*, *vga*(*A*)*LC* (*rep5*), *tet*(K) (*rep7a*), *dfrC*, *aac6′-aph2″*, *ant4′* (*rep22*, *rep20*), *fosB*	7	*qacA* (*rep22*, *rep20*)	None	GyrA (S80F)	None	ISSau4
X3617	*S. epidermidis*	H-DO/19	ST59/CC2	None	*blaZ*, *ermA*, *ant9′*, *fosB*, *mupA*	5	None	*rep7a*	None	Tn*554* (*ant9′*, *ermA*)	None
X6049b	*S. epidermidis*	H-DO/26	ST35/CC5	V	*blaZ*, *mecA msrA*, *mphC*, *tet*(K) (*rep20*), *fosB*, *fusB*, *mupA*	7	None	None	L3 (I188V, G218V, N219I, L220D) and L4 (N158S)	none	None
X6293	*S. epidermidis*	H-DO/44	ST297	II	*blaZ*, *mecA*, *ermC*, *tet*(L), *tet*(45), *dfrC*, *ant4′*, *bleO*, *fosB*	6	*qacC*, *qacJ*, *smr*	repUS22	None	None	ISSep3
X4430	*S. epidermidis*	NS/487	ST595	None	*blaZ* (*rep20*, *repUS70*), *msrA (rep20*, *repUS70)*, *fosB*, *mupA*	4	*qacC* (*rep13*), *smr*	*rep7a*, *rep39*, *rep40*	None	None	None
X4638	*S. lentus*	NS/507	NTs	Hybrid VII	*blaZ*, *mecA*, *mecC*, *mphC*, *tet*(K) (*rep7a*), *fosD*	5	None	None	None	None	None
X3574	*S. hominis*	H–D/8	ST33	VI (4B)	*blaZ*, *mecA*, *msrA*, *mphC*, *ant4′* (*rep22*), *bleO* (*rep22*), *fusC*	4	*qacA*	*rep20*, *rep21*, *rep39*	None	None	ISSau4
X4592	*S. arlettae*	NS/535	NT	None	*bla*_*ARL*_, *lnuA*, *mphC*, *msrA*, *tet*(K) (*rep7a*), *aph2′*	5	*qacG* (*rep21*)	*rep16*	GyrA (S84L)	None	None
X4956	*S. pasteuri*	H-P*/*A-P8	NT	Vc	*blaZ*, *mecA*, *ermC* (*rep10*), *vga*(*A*)*LC*, *tet*(K), *tet*(L) (*rep22*), *tet*(M)*, tet*(45), *dfrK* (*rep22*), *ant4′* (*rep22*), *str* (*rep7a*)	5	*czrC*	*rep21*, *rep39*	None	None	None
X5069	*S. hyicus*	H-P*/*C-P1	NT	None	*blaZ*, *ermT*, *lnuB*, *lsaE*, *tet*(L), *tet*(45), *dfrK*, *ant6′*	6	None	None	None	Tn*559* (*dfrK*)	None
X5447	*S. hyicus*	H-P*/*B-P9	NT	None	*blaZ*, *ermT*, *lnuB*, *lsaE*, *tet*(L), *tet*(45), *aac6′-aph2″*, *ant4′*, *ant6′*	6	None	*rep22*	None	None	IS256
X5777	*S. simulans*	H-P/C-P2	NT	None	*blaZ*, *ermA*, *ant9′*	4	None	*rep7a*, *rep21*	GyrA (E214T, V248E, S63P, Q6E, Y366R, A367T, S173A, C377H, S376V, I368V, L191V, A169V, K364R, S16N, A32S, K200H, L188M, N153T, S158E, S245A, L4Y)	Tn*559* (*ermA, ant9′*)	None

Unusual AMR determinants in bold; *NS* nestling stork, *H-P* healthy pig, *H-PF* healthy pig farmer, *H–D* healthy dog, *H-DO* healthy dog owner, *HH*^−^ healthy human without animal contact

The pigs (10 per farm) are named P1-P10 in each farm (A–D). In the case of humans working on the farm, they are designated as F1, F2, F3 and the farm (A–D)

^*^All strains were of nasal origin, except *S. haemolyticus* X3784 of nestling stork which was from tracheal sample

Concerning the genetic lineages of *S. epidermidis*, STs belonging to the clonal complexes CC2 and CC5 were identified. For the *S. haemolyticus,* the three isolates were of the lineage ST30 and ST68. Moreover, *S. sciuri*-ST212 and *S. hominis*-ST33 were detected. The genetic lineages of other species were not identified as no MLST scheme has been developed and validated for them yet.

Multiple virulence genes that mediate host immune evasion, adhesion, and haemolysis among others were identified (Table 1). It is important to remark on the detection of an *S. epidermidis* strain that carries the enterotoxin genes, *sec* and *sel.*

### Relatedness of the coagulase-negative staphylococci strains

**Fig. 1:**
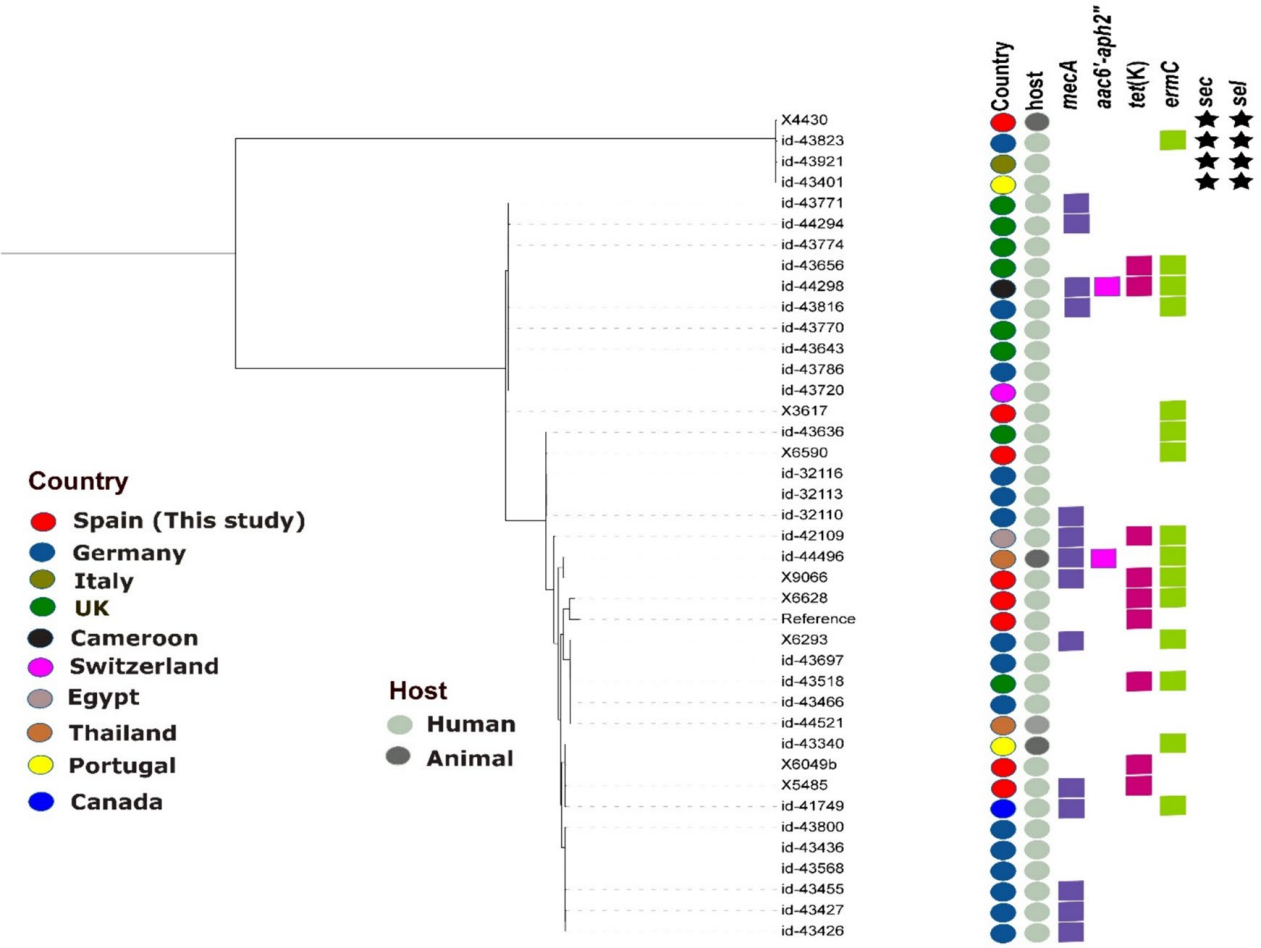
Phylogenetic tree based on core genome SNP analysis of eight *S. epidermidis* isolates of this study with 31 publicly available genomes with similar lineages

The phylogenetic analysis identified clusters of related strains of various CoNS species with other countries. Specifically, the *cfr*-carrying *S. epidermidis-*ST16 strain (X5485) was related to an *S. epidermidis-*ST16 strain from a human blood sample (SNP = 70) from Canada (id-41749). The *S. epidermidis*-ST35 from a dog owner is related to an human strain from Portugal (id-43340) (SNP = 90). Moreover, the *S. epidermidis*-ST297 from a healthy human in our study is related to three human strains from Germany, the UK, and Switzerland (SNP < 80**) (**Fig. 1). Also, the *S. epidermidis*-ST173 strain (X9066) was related to an animal strain in Thailand (id-44496) (SNP = 76) (Fig. 1). Furthermore, the *sec/sel* carrying-*S. epidermidis*-ST595 strain (X4430) is not related (> 3000 SNPs) to previously described *sec/sel*-carrying strains from Portugal and Italy (ID-43921, Id-43401). It is important to remark that despite the few SNP differences (< 85 SNPs) between some strains from Portugal (id-43340) and Canada (id-41749) with our two linezolid-resistant strains (X5485 and X6049b), none of them from the two countries was linezolid-resistant. This suggests that our strains might have acquired the gene and mutation following antibiotic pressure in the livestock niche. These findings highlight the international circulation of related *S. epidermidis* strains between humans and animals as confirmed by the phylogenetic analysis (SNP < 100) (Fig. 1**, **Supplementary Table S2).

**Fig. 2:**
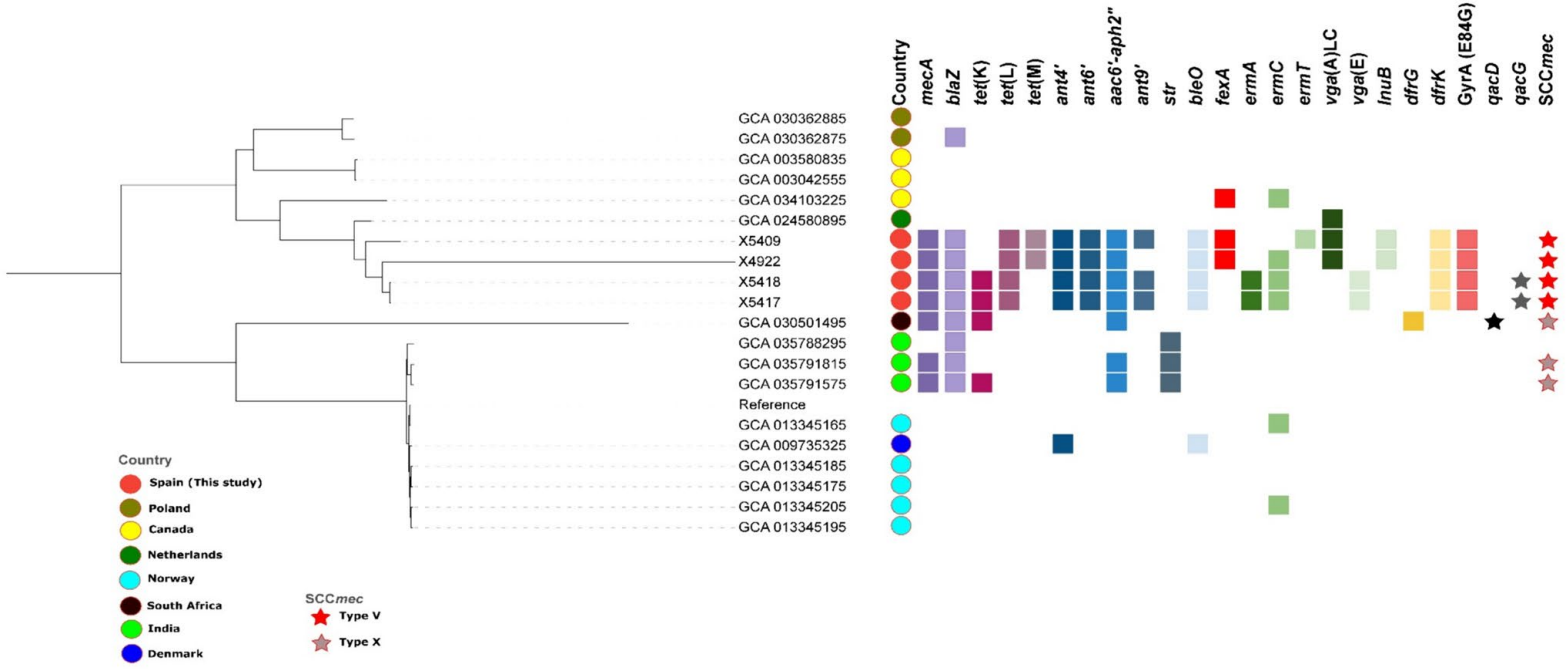
SNIP-based phylogenetic tree of the four *S. borealis* isolates of this study mapped with all the 16 publicly available genomes

Aside from the *S. epidermidis* strains, we found related *S. borealis* (SNP < 10) between pigs (Fig. 2**, **Supplementary Table S3**)**. However, the relatively high SNP (*n* = 346) between the *S. saprophyticus* strains from a pig and pig farmer on the same farm suggests that they are unlikely related **(**Supplementary Table S4).

### Mobilome-bound antimicrobial resistance in coagulase-negative staphylococci

Generally, MDR was the criteria of selection, and so, all isolates need to be resistant to at least 4 classes of antibiotics **(**Supplementary Table S1**)**. In this sense, the resistome profile of the strains was mainly to beta-lactam, macrolide-lincosamide-streptogramin-B (MLS_B_), tetracyclines, sulfamethoxazole-trimethoprim, aminoglycosides, and phenicols as previously detected by PCR and presented in our previous study (Abdullahi et al. 2023a). MDR to a maximum of 5 antibiotic classes was found from the previous study on CoNS strains from nestling storks (NS) (Abdullahi et al. 2023a). For the healthy dogs (H–D), healthy dog owners (H-DO), pigs (H-P), and pig farmers (H-PF), resistance to a maximum of nine antibiotic classes was obtained. In the case of isolates of  healthy humans without animal contact (HH^−^) resistance to a maximum of seven antibiotic classes were obtained. Resistance genes already detected by PCR were found (Abdullahi et al. 2023a, b, c, 2024a, b, c), but others not previously identified such as *lsaB*, *lsaE*, *vgaA*(*LC*), *vga*(*E*), *bleO*, *str*, and *dfrC* were identified (Table 1). Concerning plasmid bound-AMR genes, all the MRCoNS from pigs and pig farmers had *mecA* genes carried by SCC*mec* type-Vc except the two *S. saprophyticus* strains that had *mecA* in SCC*mec* type-IVb. The predominance of the SCC*mec* type-Vc in these isolates strongly suggests the interspecies transmission of *mecA* gene by the same SCC*mec* element. Thus, it has been speculated that the SCC*mec* type-Vc in LA-MRSA originated from MR-CoNS carried in the same ecological niche (such as nostrils in this case) (Matuszewska et al. 2022). This is corroborated by the SCC*mec* types carried by LA-MRSA-CC398 isolates from the same pigs and pig farmers (Abdullahi et al. 2024b). Whereas the SCC*mec* type-IV (a common community SCC*mec* type in MRSA) in *S. saprophyticus* from the pig and pig farmer suggests community-associated strains brought to the pig farm. Moreover, *S. saprophyticus* is known to cause uncomplicated urinary tract infections in the community (Lawal et al. 2021a, b). In nestling storks, the MDR-*S. arlettae* and *S. epidermidis* isolates were methicillin-susceptible, whereas the MR-*S. haemolyticus* carried *mecA* gene located in SCC*mec* type-V. Moreover, the MR-*S. lentus* carried the *mecA/mecC* genes located in SCC*mec-mecC* hybrid. It is important to remark that the *mecA* gene might be intrinsic in *S. lentus* (Saber et al. 2017). Of the MR-CoNS strains from dogs/owners and healthy humans, both the classical hospital and community-associated SCC*mec* elements were detected. This shows the SCC*mec* type in these hosts aside from pigs/farmers has no categorical predilection.

Concerning genes that encode MLS_B_ resistance, the *ermB*, *ermC*, *erm45*, *vgaA*(*LC*), and *vga*(*E*) genes were identified in single or in combination among over 50% of the CoNS isolates (Table 1). Specifically, the *ermC* gene in most of the *ermC*-positive strains was located in small plasmids that were 99.8% identical to those previously described in an *S. aureus* isolate, plasmid pMSA16 (GenBank accession number: JQ246438.1) and in an *S. saprophyticus* isolate, pSES22 (GenBank accession number: AM159501.1). Moreover, it is important to remark on the detection of the unusual *ermT* gene in two staphylococcal species: *S. borealis* (carried by plasmid *repUS18*) and *S. hyicus* (with no associated plasmid). The *ermT* gene is not a common mechanism for MLS_B_ resistance in CoNS. It appears *ermT* gene is silently evolving in CoNS causing a constitutive MLS_B_ resistance phenotype.

Tetracycline resistance was found in all the pigs’ and pig farmers’ isolates and mediated by different combinations of genes. In this regard, *tet*(K), *tet*(L), *tet*(M), and *tet*(45) were found in most of the pigs/pig farmers isolates **(**Table 1**)**. Moreover, the *tet*(L) gene was found in one *S. epidermidis* (X6293) isolate from a dog owner. It is important to mention that the *tet*(L) gene was located in plasmid *rep22* in all the pigs and farmers isolates. However, no MGE was detected to be associated with all the *tet*(M) and *tet*(45) carrying MDR-CoNS strains. The absence of MGE associated with *tet*(M) gene in the MDR-CoNS is different from the transposon-linked *tet*(M) gene found in the *S. aureus* strains (Abdullahi et al. 2024b), and this is subject to further investigations to unravel the reasons for the differences. Perhaps, this plasmid *rep22*-located *tet*(L) gene is coincidentally predominant in the pig farm niche. The *tet*(K) gene in most of the CoNS isolates was located in *rep7a* while in only one strain (X6049b) was located in plasmid *rep20,* and three others from pigs were not associated with this plasmid replicon (Table 1). It is important to highlight that all the plasmid bound-*tet*(L) genes were linked with the *dfrK* gene in similar plasmid *rep*US12. A similar observation was reported in an MRSA-CC398 strain from a pig (GenBank accession number: FM207105). However, *tet*(L) was not found to be located in any plasmid in one of the *S. hyicus* strains from a pig (X5069) carrying a Tn*559*-bound *dfrK.* This denotes the difference in the pattern of acquisition of *tet*(L) gene and potential inter-species transmission in CoNS and *S. aureus* in a pig farm setting.

Aside from these plasmid-bound AMR genes, other genes that mediate resistance to aminoglycosides (such as *ant4′* and *bleO*, located in plasmid *repUS12*), clindamycin (e.g., *lnuA*, in *rep22*), and sulfamethoxazole-trimethoprim (e.g., *dfrK*, in *repUS12* and *rep22*) were occasionally identified. In some instances, these AMR genes were not associated with any plasmid. We cannot categorically infer the reason some AMR genes are located in plasmids in some CoNS strains while in the bacterial chromosome of others. It could be that the bacteria lost the plasmids during horizontal transfer but the recipient bacteria retained the AMR genes (Dimitriu 2022). The similarity in plasmids that carry many AMR genes in all the CoNS strains demonstrates their impact on bacterial fitness for survival and capability to transfer these resistant genes intra-species (the same species), interspecies, and between different hosts. The transferability of AMR genes between different *Staphylococcus* species has been strongly suggested by the sequence similarity of their associated mobilome, especially plasmids (Souza-Silva et al. 2022). Moreover, some plasmids appeared to carry multiple AMR genes from different classes of antibiotics (such as *repUS12* and *rep22*).

Aside from these mobilome-bound AMR genes, the aminoglycoside and MLS_B_ resistance genes *ant9′* and *ermA* were also carried by Tn*554* in an *S. epidermidis* strain from a dog owner (X3617). Similar findings (i.e., Tn*544*-linked *ant9′* and *ermA* genes) was reported but in a different CoNS species, *S. lugdunensis* (Chang et al. 2019, 2021). This suggests potential inter-staphylococcal species transmission of the ARGs. Chloramphenicol resistance is an important phenotypic marker for linezolid resistance, especially in pig farm settings. Chloramphenicol has long been prohibited for the treatment of animal and human infections in Spain. However, florfenicol is still used for livestock. The *fexA* and *fexB* genes confer resistance to both florfenicol and chloramphenicol and could be responsible for the frequent co-resistance to chloramphenicol found in CoNS strains from pigs and pig farmers. In this study, only *fexA* which was carried by *Tn554* and *Tn558* was identified in four pigs’ strains and this illustrates the influence of pig farm setting on the persistence of phenicol resistance genes especially the *fexA* that could be carried by two different transposons. Of clinical and public health concern is that other critical AMR genes such as those that mediate transferable linezolid resistance could be co-selected. In this regard, two *cfr*-carrying *S. epidermidis* and *S. saprophyticus* isolates from a pig previously identified were identified (Abdullahi et al. 2023b). Upon genomic characterization, the *cfr* gene in *S. saprophyticus* strain was located in a plasmid *rep15*, while in *S. epidermidis* was not associated with any plasmid but was flanked by ISSau9 (Table 1).

### Antimicrobial resistance mediated by chromosomal point mutations

Twelve of the 26 CoNS isolates analyzed (46.2%) carried one or more mechanisms of ciprofloxacin resistance mediated by DNA topoisomerase IV point mutations at GyrA (S84L) and DNA gyrase at GrlA (S80F) **(**Table 1**)**. Interestingly was the detection of 21 non-synchronous mutations on the GyrA on one *S. simulans* strain from a healthy pig (X5777) **(**Table 1**)**. A major difference in the ciprofloxacin resistance rate was observed between the isolates from the pigs and pig farmers and those of the other hosts: 7 (50%) of the CoNS isolates from pigs and pig farmers showed one or more of the mutations on quinolone-resistance-determining region, whereas three CoNS isolates from healthy humans (*S. epidermidis* and *S. haemolyticus*) and one *S. arlettae* isolate from a nestling stork exhibited this mutation **(**Table 1**)**. These highlight the influence of pig farm antibiotic pressure on ciprofloxacin resistance on the CoNS isolates. Moreover, mutation-mediated AMR related to linezolid resistance was found in ribosomal proteins L3, L4, and L22 in a *S. epidermidis*-ST15 strain from a dog owner, as previously identified by PCR-sequencing (Abdullahi et al. 2023c).

### Plasmid-bound biocide and metal resistance among the CoNS isolates

Concerning biocide resistance, various plasmid bound-biocide resistance genes (such as *qacA* [*rep20*, *rep22*], *qacC* [*rep13*], *qacG* [*rep21*], and *qacJ* [*rep21*]) were detected in 34.6% of the 26 MDR-CoNS isolates characterized in this study. The acquisition of *qacG* gene carried on plasmid *rep*21 was previously found in the majority of *S. aureus* strains from our previous study (Abdullahi et al. 2024b). This plasmid-bound resistance to quaternary ammonium compounds could facilitate the persistence and co-selection of MDR in CoNS, as these genes make it very difficult for their eradication (Seier-Petersen et al. 2015). In addition, *smr* gene that encodes resistance against cationic antiseptic compounds (Damavandi et al. 2017) was identified in four strains (Table 1). Two genes that encode for cadmium and zinc resistance (*cadD* and *czrC, *respectively*) *were identified, of which *czrC* predominated (42.3%).

Metal resistance has previously been hypothesized to co-select for AMR and they are often linked to SCC*mec* elements (Lawal et al. 2021b) and plasmids in LA-MRSA, *S. epidermidis*, *S. saprophyticus*, *S. haemolyticus*, etc. (Lawal et al. 2021a; Argudín and Butaye 2016; Schijffelen et al. 2010). Specifically, determinants of copper (*copA*) and zinc (*czrC*) resistance were widespread among our MR-CoNS isolates of the pigs and pig farmers, but absent or minimal in other hosts. This could denote the potential selection of resistance to these metals due to their persistence in pig farm settings (e.g., in pig feed) especially when plasmid-linked (Huang et al. 2021; Slifierz et al. 2015). Moreover, the cadmium resistance gene (*cadD*) suggests the involvement of environmental pollution where these staphylococci originated (Rebelo et al. 2021).

### Genetic environment of the unusual antimicrobial resistance gene in CoNS strains

**Fig. 3:**
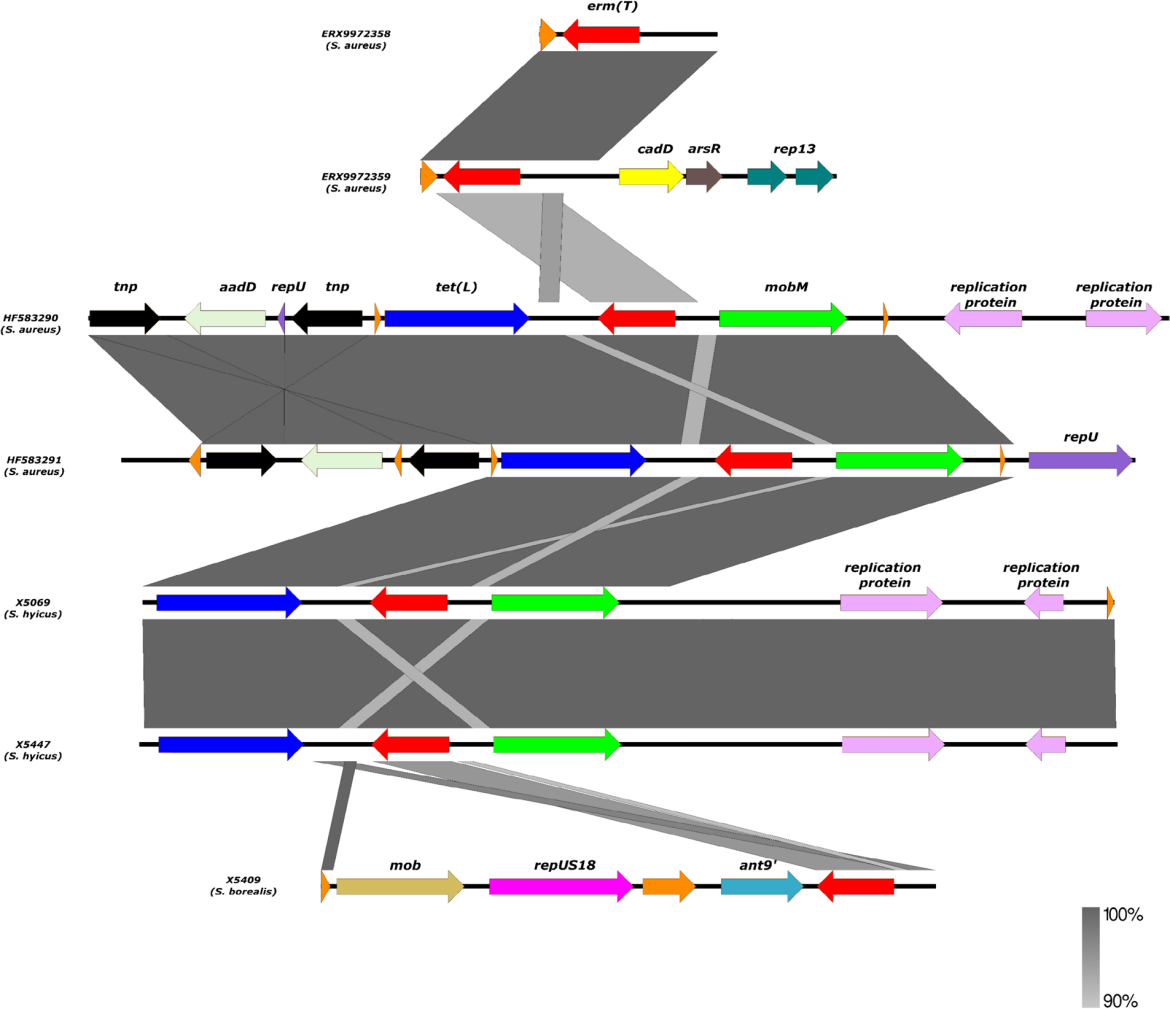
Genetic environment of *ermT* gene of three CoNS isolates of this study (X5447, X5069, and X5409) in comparison with those of four reference strains

The in silico analysis of the *ermT* sequences of three CoNS strains of two different species (*S. borealis* and *S. hyicus*) from healthy pigs revealed major differences in their genetic environment (Fig. 3). The *ermT* gene is in the opposite direction respect to a*nt9′* and both are located in plasmid *repUS18* in *S. borealis* strain. However, the *ermT* gene in the other two *S. hyicus* strains (X5447 and X5069) is not associated with any plasmid, perhaps it is chromosomally located. The *ermT* gene in the three strains produces an erythromycin-clindamycin resistance phenotype of constitutive character and highlights their evolution in MLS_B_ resistance among CoNS.

**Fig. 4:**
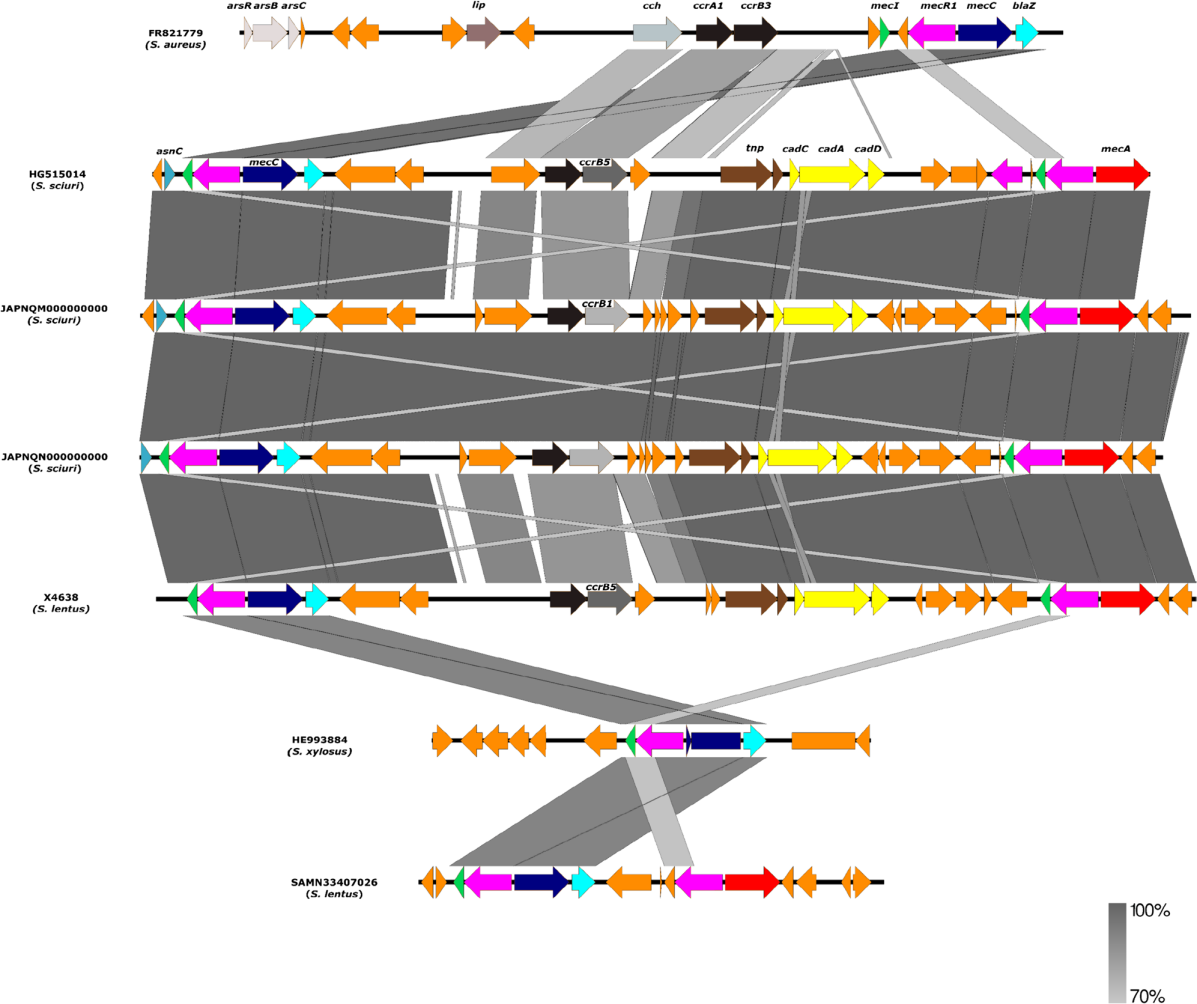
The environment of the *mecC* gene of *S. lentus* (X4638) in comparison with previously described *mecC*-carrying coagulase-negative staphylococci and the *S. aureus*_LGA251_ strain

The in silico analysis of *S. lentus* strain (X4638) showed that it carried a hybrid SCC*mec-mecC*, which is 100% similar to an *S. sciuri* strain from bovine infection in the UK (Harrison et al. 2014). Specifically, the SCC*mec*-*mecC* hybrid consisted of a class C1 *mec* complex located immediately downstream of a SCC*mec* type-VII element. Moreover, the *cadA*, *cadC*, and *cadD* genes are included in the system **(**Fig. 4**).** It has been previously described that most CoNS that carry the *mecC* gene are within a hybrid SCC*mec* element comprised of *mecA* included in SCC*mec* type VII and a *mecC* region consisting of the class E *mec* complex (de Moura et al. 2023; Belhout et al. 2023; Paterson 2020). However, *blaZ-*SCC*mec* XI was initially found to be associated with *mecC* in our *S. lentus* X4630 strain by PCR and amplicon sequencing by Sanger (Abdullahi et al. 2023a). Following WGS, the *mecC* gene of the *S. lentus* X4638 strain was noted to be quite different from the classical SCC*mec* type XI that was first demonstrated in *S. aureus*_LGA251_ (accession number FR821779). The reason for this variation is subject of further analysis. But, it could be hypothesized that a recombination event took place between the SCC*mec* type III (intrinsic for most MR-*S. lentus*) of the *mecA* gene and SCC*mec* type XI of the *mecC* to produce the SCC*mec-mecC* hybrid (i.e., the SCC*mec* type VII). In this regard, there is a need for caution in the use of PCR-based assays for the detection of SCC*mec* types in *mecC*-carrying non-*aureus* staphylococci. To the best of our knowledge, this report represents the first description of a *mecC* in an *S. lentus* strain from a wild bird. This suggests the expansion of this mechanism of methicillin resistance in CoNS across various ecological niches including wild animals, which were previously proposed to be the major reservoirs of the *mecC* gene in *S. aureus* (Abdullahi et al. 2021).

**Fig. 5:**
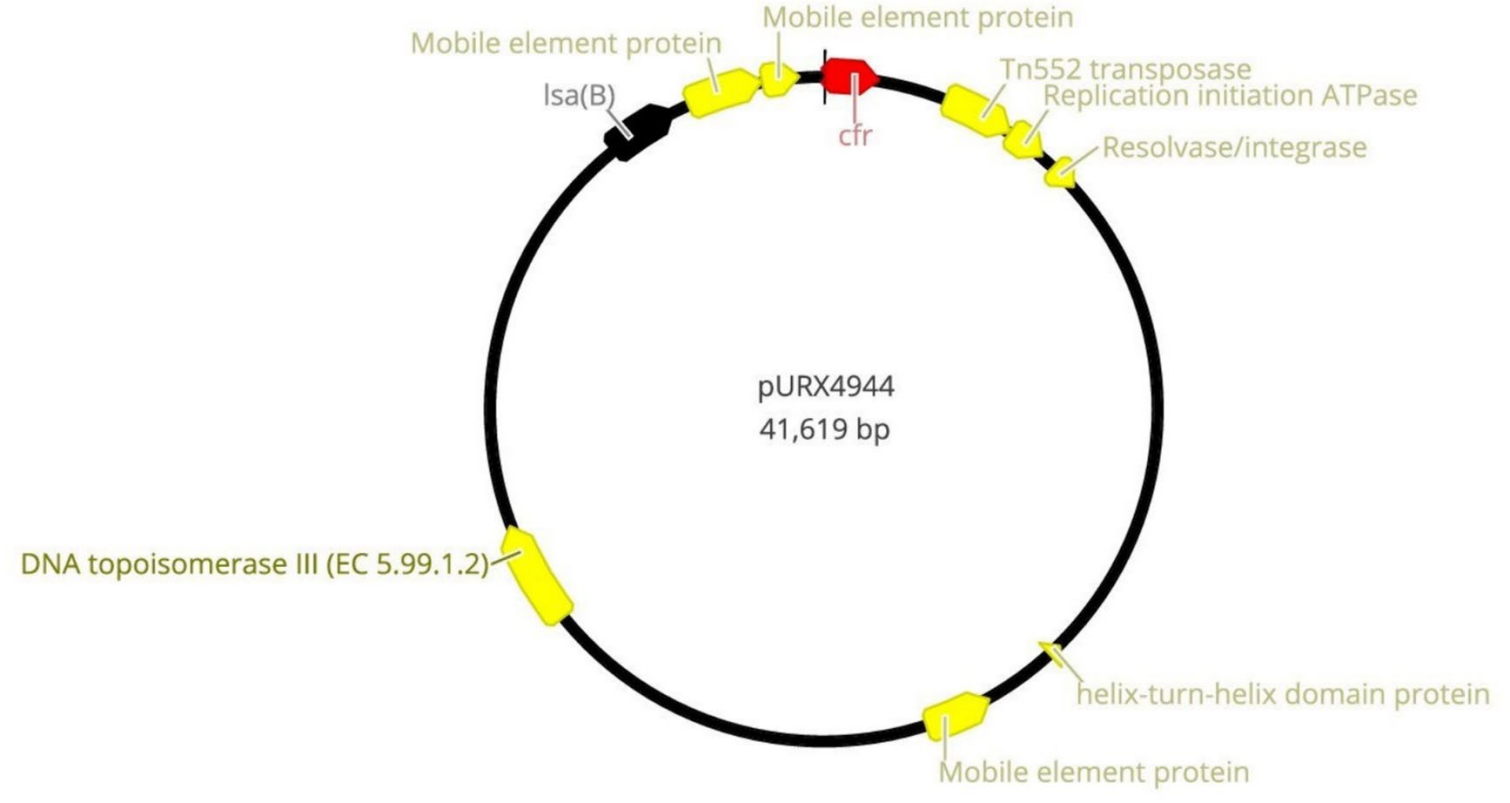
Circular representation of the plasmid-carrying the *cfr* gene in *S. saprophyticus*

**Fig. 6:**
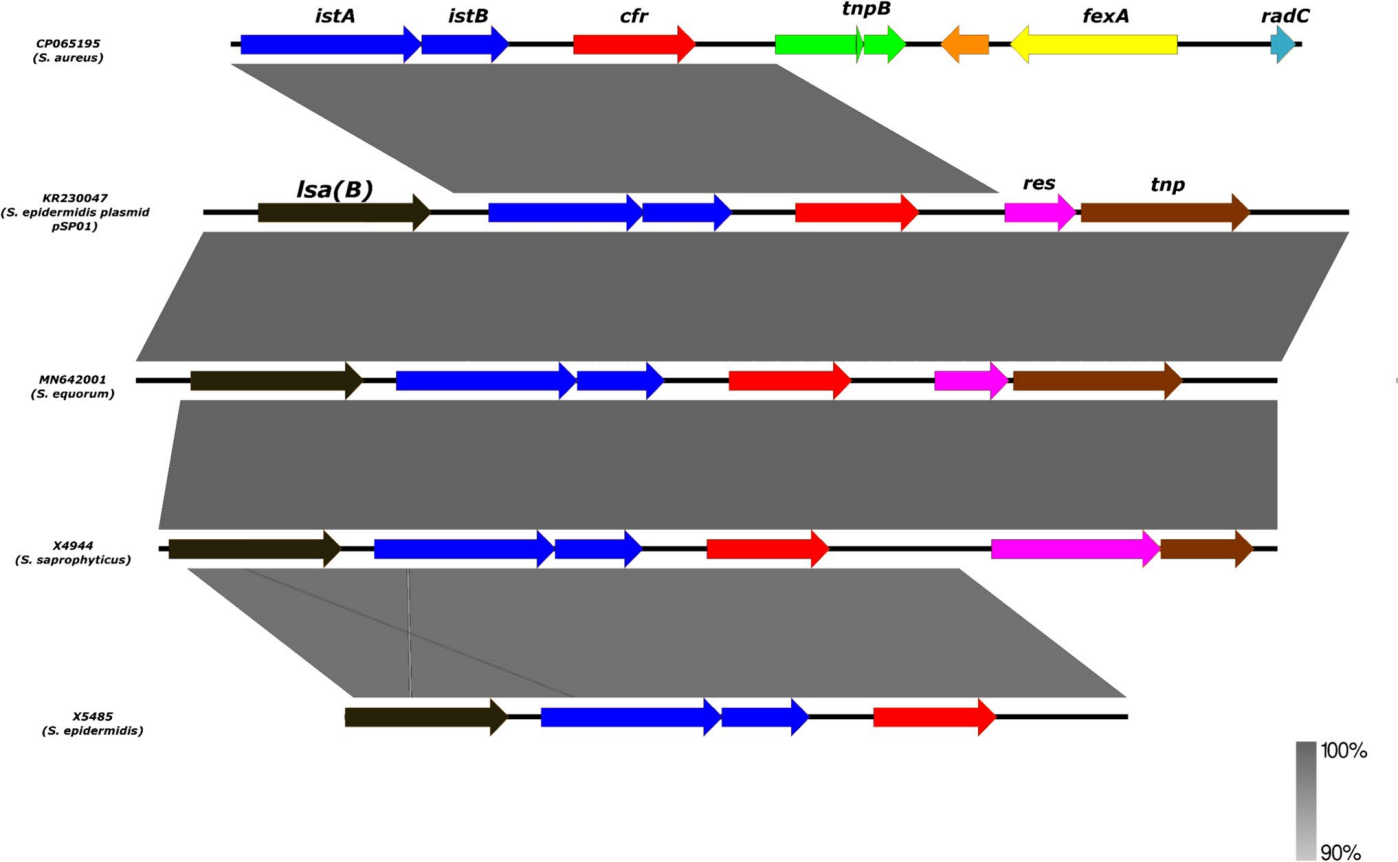
Environment of the *cfr* gene of *S. epidermidis* (X5485) and *S. saprophyticus* (X4944) in comparison with previously described *cfr*-carrying coagulase-negative staphylococci and *S. aureus*

The linezolid resistance mechanism mediated by plasmid pURX4944 (41.6 Kb) (Fig. 5) carrying the *cfr* gene located upstream of *lsaB* was identified in *S. saprophyticus* X4944 strain and it was 96% identical to the plasmid of a clinical *S. epidermidis* strain from Italy (GenBank accession number: KR230047.1). Nevertheless, the *cfr* gene of our *S. epidermidis*-ST16 strain was not associated with a plasmid but was flanked by IS256 upstream of *lsaB* (Fig. 6)*.* It has been suggested that the emergence and dissemination of the *cfr* gene in animals that have never used any of the oxazolidinones might be due to the selective pressure by the high use of florfenicols, lincosamides, tetracyclines, and pleuromutilins in the livestock sector (Gostev et al. 2021).

### Virulome profile of the coagulase-negative staphylococci strains

We investigated the frequency and distribution of virulence genes among the different CoNS isolates from the four hosts. About 96.1% of the MDR-CoNS strains harbored one or more of diverse adherence, exoenzymes, haemolysin, or immune evasion genes (Table 2). Enterotoxins constitute important virulence determinants of the genus *Staphylococcus*, of which they are rarely detected in CoNS (França et al. 2021). Enterotoxins are the most implicated in food-borne gastroenteritis (Grispoldi et al. 2021). Moreover, other virulence factors could be responsible for a range of staphylococcal-related infections that are rarely detected in non-*aureus* staphylococci (Nanoukon et al. 2018). However, it is important to highlight the detection of a *sec*- and *sel*-carrying *S. epidermidis* strain of the lineage ST595. Similar studies have previously reported these virulence genes and their associated pathogenicity islands in *S. epidermidis* (Lin et al. 2021; Nasaj et al. 2020; Banaszkiewicz et al. 2019). Moreover, it has been suggested that only *S. epidermidis* from animals or food but not from humans may typically produce *S. aureus-*related enterotoxins (Podkowik et al. 2016; Veras et al. 2008; Stach et al. 2015; Nanoukon et al. 2018). However, some *sec* and *sel* genes have been identified in association with plasmids, phages, and pathogenicity islands. Thus, they can be horizontally transmitted between any host, including humans. It appears that the *sec* and *sel*-carrying *S. epidermidis* from nestling stork are not transferable as they were not associated with a mobile genetic element. Moreover, simultaneous colonization of the nostril by several *Staphylococcus* spp could promote the transfer of enterotoxin genes from *S. aureus* to commensal *S. epidermidis* (Nanoukon et al. 2018).

**Table 2:** Virulence determinants and prophages in the 26 MDR-CoNS isolates

Strain ID	Species	Source/ID^a^	Intact staphylococcal phages identified (GenBank accession number)	Classes of virulence factors						
				Adherence	Exoenzymes	Haemolysin	Immune evasion	Mobile genetic element	Metal uptake	others
X4922	*S. borealis*	H-P/A-P8	IME_SA4 (NC_029025)	*atl, ebp*	*lip*	None	*adsA*	None	None	None
X5417	*S. borealis*	H-P/B-P4	vB_SepiS-phiIPLA5 (NC_018281)	*atl*, *epb*	*lip*	None	*adsA, wbtP*	None	None	*uge*
X5418	*S. borealis*	H-P/B-P5	vB_SepiS-phiIPLA5 (NC_018281)	*atl, epb*	*lip*	None	*adsA, wbtP*	None	None	*uge*
X5409	*S. borealis*	H-P*/*B-P4	IME_SA4 (NC_029025)	*atl*, *epb*, *sdrE*	*lip*	None	*adsA*, *capB*, *wbtP*	None	None	None
X4944	*S. saprophyticus*	H-P/A-P10	47 (NC_007054)	*atl*, *sdrC*	*lip*, *sspA*	None	None	None	None	None
X5435	*S. saprophyticus*	H-P*/*B-P6	phiRS7 (NC_022914)	*atl*, *ebp*	*lip*, *geh*	None	*capB*, *galE*	None	*vctC*	None
X5462	*S. saprophyticus*	H-PF*/*B-F1	phiRS7 (NC_022914)	*atl*	*lip*, *geh*, *sspA*	None	*capB*, *galE*	None	*vctC*	None
X5776	*S. haemolyticus*	H-PF/D-F2	stB12 (NC_020490)	None	*sspB*, *geh*	None	*adsA*, *capB*, *galE*, *wbtE*, *wbtP*	None	None	*cylR2*
X7059	*S. haemolyticus*	HH/34	stB12 (NC_020490)	*atl*, *ebp*	*lip*	None	None	None	None	None
^*^X3784	*S. haemolyticus*	NS/546	None	*atl*, *epb*	*lip*	None	*adsA*, *wbtP*	None	None	None
X4892	*S. sciuri*	H-P/A-P2	None	*icaA*, *icaB*, *icaC*, *clpP*, *lgt*	*sspA*	None	None	None	*vctC*	*lisR*
X5485	*S. epidermidis*	H-PF/B-F1	StB20 (NC_019915)	*icaA, icaB, icaC, icaR, sdrH, sdrG, atl, ebh, ebp*	*sspA, sspB, lip, geh*	*hlb*	None	ACME	None	None
X6590	*S. epidermidis*	HH/19	None	*atl*, *ebh*, *ebp*, *sdrH*, *geh*	*sspA*, *sspB*, *lip*	*hlb*	None	ACME	None	None
X6628a	*S. epidermidis*	HH/22	None	*sdrH*, *sdrG*, *atl*, *ebh*, *ebp*	*sspA*, *sspB*, *lip*, *geh*	None	None	ACME	None	None
X9066	*S. epidermidis*	HH/46	None	*atl*, *ebp*, *ebh*, *clfA*, *icaA*, *icaB*, *icaC*, *icaR*, *sdrD*, *sdrG*	*sspB*, *geh*, *lip*, *sspA*	*hlb*	*capB*	ACME	None	None
X3617	*S. epidermidis*	H-DO/19	None	*sdrH*, *sdrG*, *atl*, *ebh*, *ebp,*	*sspA*, *sspB*, *lip*, *geh*	*hlb*	None	ACME	None	None
X6049b	*S. epidermidis*	H-DO/26	stB12 (NC_020490)	*icaA*, *icaB*, *icaC*, *icaR*, *sdrH*, *sdrG*, *atl*, *ebh*, *ebp*, *eno*	*sspA*, *sspB*, *lip*, *geh*	*hlb*	None	ACME	None	None
X6293	*S. epidermidis*	H-DO/44	None	*icaA*, *icaB*, *icaC*, *icaR*, *sdrF*, *sdrG*, *sdrH*, *atl*, *ebh*, *ebp*	*sspA*, *sspB*, *lip*, *geh*	*hlb*	None	ACME	None	None
X4430	*S. epidermidis*	NS/487	None	*alt*, *ebh*, *epb*, *sdrG*	*lip*, *sspB*, *geh*, *esa*, *esaD*, *esaE*, *esaG*, *essB*, *essC*, *esxB*, *esxC*, *esxD*	*hlb*	None	None	None	***sec, sel***
X4638	*S. lentus*	NS/507	None	*clfB*, *lgt*	*sspA*, *ndk*, *lplA1*	None	*gtaB*, *wbtP*	None	*vctC*, *ctpV,*	*lisR*
X3574	*S. hominis*	H–D/8	stB_27 (NC_019914)	*atl*, *ebp*	*lip*	None	*capB*	None	None	None
X4592	*S. arlettae*	NS/535	vB_SauS_phi2 (NC_028862)	None	*lip*, *sspA*	None	*gale*, *wbtP*	None	None	None
X4956	*S. pasteuri*	H-P*/*A-P8	vB_SepiS-phiIPLA7 (NC_018284)	*atl*, *ebh*, *ebp*, *icaA*, *icaB*, *icaC*	*lip*, *sspB*	None	*capB*, *manA*	None	None	None
X5069	*S. hyicus*	H-P*/*C-P1	EW (NC_007056)	*clfA*, *clfB*, *cna*, *fnbA*, *fnbB*	*sspB*, *hysA*, *geh*, *esaB*, *essB*, *essC*, *esxA*	*hlb*	*capB*, *capC*	None	None	*set26*, *cvtC*, *lisR*, *lgt*
X5447	*S. hyicus*	H-P*/*B-P9	None	*clfA*, *cna*, *fnbA*, *fnbB*, *lgt*	*sspB*, *hysA*, *geh*, *esaB*, *essB*, *essC*, *esxA*	*hlb*	*adsA,capB*, *capC*	None	*vctC*	*set15*, *lisR*
X5777	*S. simulans*	H-PC-P2	37 (NC_007055)	*atl*, *ebp*	None	None	*capB*	None	None	None

*uge*, antiphagocytic capsule; *lisR*, signal transduction system; unusual virulence genes in bold

^a^*NS* nestling stork, *H-P* healthy pig, *H-PF* healthy pig farmer, *H–D* healthy dog, *H-DO* healthy dog owner, *HH*^−^ healthy human without animal contact

The pigs (10 per farm) are named P1-P10 in each farm (A–D). In the case of humans working on the farm, they are designated as F1, F2, F3 and the farm (A–D)

^*^All strains were of nasal origin, except *S. haemolyticus* X3784 of nestling stork which was from a tracheal sample

Aside from the toxins, many CoNS harbored genes such as the *capB* and *capC* (encode capsules) and *adsA*, *galE*, *wbtE*, *wbtP* genes that facilitate immune evasion by CoNS (Naushad et al. 2019; Li et al. 2015). Furthermore, the *icaABC* operon and its *icaR* were present in five strains (19.2%) (Table 2). This denotes that some of the CoNS species could easily adhere to the mucosa and inanimate surfaces and serve as a fundamental step in colonization and persistence on environmental surfaces and fomites (Idrees et al. 2021).

### CRISPR-Cas system distribution among the coagulase-negative staphylococci

Complete CRISPR-Cas system was detected in 19.2% of the CoNS strains, of which *cas-*1, -2, and -9 predominated in *S. borealis* (75%). In other species, Cas3-type I CRISPR was identified in two *S. epidermidis* strains (X6590 and X6049b) from humans. Furthermore, the *mecC*-carrying *S. lentus* harbored Cas2-type I and Cas9-type II (Table 3). The low frequency of CRISPR-Cas positive strains identified in our study is closely similar to the 12.3% rate by Rossi et al. (2017) which consisted of mainly class 1 type IIIA and class 2 type IIC systems. Considering that most CRISPR-Cas reduces or eliminates mobile genetic elements such as plasmids, the low frequency of CRISPR-Cas elements among MDR-CoNS isolates in this study could explain the reason why all the strains had ARGs carried by multiple plasmids. However, large-scale genome-based studies including isolates with different profiles of antibiotic resistance are necessary to better understand the roles of the CRISPR-Cas system on AMR genes and their plasmids among *S. borealis*.

**Table 3:** CRISPR-Cas system distribution among the CoNS isolates

Strain ID	Species	Source/ID^a^	No. of standalone CRISPR/No. of with Cas protein	CRISPR-Cas class	Cas type (orientation)	Total number of spacers/spacers with Cas
X4922	*S. borealis*	H-P/A-P8	14/0	None	None	14
X5417	*S. borealis*	H-P/B-P4	12/3	Class 2 type II	Cas1-type II ( +); Cas2-type-I, II, III ( +); Cas9-type II ( +)	18/3
X5418	*S. borealis*	H-P/B-P5	13/3	Class 2 type II	Cas1-type II ( −); Cas2-type-I, II, III ( −); Cas9-type II ( −)	19/3
X5409	*S. borealis*	H-P*/*B-P4	18/3	Class 2 type II	Cas1-type II ( −); Cas2-type-I, II, III ( −); Cas9-type II ( −)	26/3
X4944	*S. saprophyticus*	H-P/A-P10	1/0	None	None	1
X5435	*S. saprophyticus*	H-P*/*B-P6	2/0	None	None	2
X5462	*S. saprophyticus*	H-PF*/*B-F1	1/0	None	None	1
X5776	*S. haemolyticus*	H-PFD-F2	2/2	None	None	2
X7059	*S. haemolyticus*	HH/34	3/0	None	None	3
^*^X3784	*S. haemolyticus*	NS/546	3/0	None	None	3
X4892	*S. sciuri*	H-P/A-P2	6/0	None	None	6
X5485	*S. epidermidis*	H-PF/B-F1	4/0	None	None	4
X6590	*S. epidermidis*	HH/19	5/1	None	Cas3-type I ( +)	5/1
X6628a	*S. epidermidis*	HH/22	1/0	None	None	1
X9066	*S. epidermidis*	HH/46	4/0	None	None	4
X3617	*S. epidermidis*	H-DO/19	1/0	None	None	1
X6049b	*S. epidermidis*	H-DO/26	4/1	None	Cas3-type I ( −)	1
X6293	*S. epidermidis*	H-DO/44	2/0	None	None	2
X4430	*S. epidermidis*	NS/487	4/0	None	None	4
X4638	*S. lentus*	NS/507	8/2	Class 2 type II	Cas2-type I, II, III ( +); Cas9-type II ( +)	15/3
X3574	*S. hominis*	H–D/8	3/0	None	None	3
X4592	*S. arlettae*	NS/535	None	None	None	None
X4956	*S. pasteuri*	H-P*/*A-P8	1/0	None	None	1
X5069	*S. hyicus*	H-P*/*C-P1	2/3	Class 2 type II	Cas1-type II ( −); Cas2-type-I, II, III ( −); Cas9-type II ( −)	26/3
X5447	*S. hyicus*	H-P*/*B-P9	2/0	None	None	20
X5777	*S. simulans*	H-PC-P2	1/0	None	None	1

^a^*NS* nestling stork, *H-P* healthy pig, *H-PF* healthy pig farmer, *H–D* healthy dog, *H-DO* healthy dog owner, *HH*^−^ healthy human without animal contact

The pigs (10 per farm) are named P1-P10 in each farm (A–D). In the case of humans working on the farm, they are designated as F1, F2, F3 and the farm (A–D)

^*^All strains were of nasal origin, except *S. haemolyticus* X3784 of nestling stork which was from a tracheal sample

## Conclusion

These findings showed that various healthy ecological niches harbor CoNS that presented MDR phenotype mediated by multiple ARGs with several mobile genetic elements with relatively low frequency of intact CRISPR-Cas systems. Furthermore, our findings highlight the potential geographical dissemination of some lineages of CoNS species across various hosts. Collectively, our findings underscore the need to strengthen the genomic epidemiological approach and inclusion of MDR-CoNS from all hosts to adequately control the global fight against AMR and potentially pathogenic ones as identified in the *sec-* and *sel*-carrying *S. epidermidis.*

## Supplementary Information

Below is the link to the electronic supplementary material.

Supplementary file1 (DOCX 21 KB)

Supplementary file2 (XLSX 19 KB)

Supplementary file3 (XLSX 14 KB)

Supplementary file4 (XLSX 9 KB)

## Data Availability

No datasets were generated or analysed during the current study.

## References

[CR1] Abdullahi IN, Fernández-Fernández R, Juárez-Fernández G, Martínez-Álvarez S, Eguizábal P, Zarazaga M, Lozano C, Torres C (2021) Wild animals are reservoirs and sentinels of *Staphylococcus aureus* and MRSA clones: a problem with “One Health” concern. Antibiotics (basel, Switzerland) 10(12):1556. 10.3390/antibiotics10121556 34943768 10.3390/antibiotics10121556 PMC8698730

[CR2] Abdullahi IN, Lozano C, Höfle Ú, Cardona-Cabrera T, Zarazaga M, Torres C (2023a) Antimicrobial resistome of coagulase-negative staphylococci from nasotracheal cavities of nestlings of Ciconia ciconia in Southern Spain: detection of mecC-SCCmec type-XI-carrying S. lentus. Comparative Immunol, Microbiol Infectious Dis 99:102012. 10.1016/j.cimid.2023.102012 10.1016/j.cimid.2023.102012 37453201

[CR3] Abdullahi IN, Lozano C, Simon C, Zarazaga M, Torres C (2023b) Within-host diversity of coagulase-negative staphylococci resistome from healthy pigs and pig farmers, with the detection of cfr-carrying strains and MDR-S borealis. Antibiotics 12:1505. 10.3390/antibiotics12101505 37887206 10.3390/antibiotics12101505 PMC10604674

[CR4] Abdullahi IN, Lozano C, Zarazaga M, Saidenberg ABS, Stegger M, Torres C (2023c) Clonal relatedness of coagulase-positive staphylococci among healthy dogs and dog-owners in Spain. Detection of multidrug-resistant-MSSA-CC398 and novel linezolid-resistant-MRSA-CC5. Front Microbiol 14:1121564. 10.3389/fmicb.2023.1121564 36937268 10.3389/fmicb.2023.1121564 PMC10017961

[CR5] Abdullahi IN, Juárez-Fernández G, Höfle U, Latorre-Fernández J, Cardona-Cabrera T, Mínguez-Romero D, Zarazaga M, Lozano C, Torres C (2023d) *Staphylococcus aureus* carriage in the nasotracheal cavities of white stork nestlings (Ciconia ciconia) in Spain: genetic diversity, resistomes and virulence factors. Microb Ecol. 10.1007/s00248-023-02208-8s 36964230 10.1007/s00248-023-02208-8 PMC10497646

[CR6] Abdullahi IN, Lozano C, González-Azcona C, Zarazaga M, Torres C (2024a) Genetic diversification and resistome of coagulase-negative staphylococci from nostrils of healthy dogs and dog-owners in La Rioja, Spain. Pathogens (basel, Switzerland) 13(3):229. 10.3390/pathogens13030229 38535572 10.3390/pathogens13030229 PMC10974962

[CR7] Abdullahi IN, Lozano C, González-Azcona C, Zarazaga M, Torres C (2024b) Genetic diversification and resistome of coagulase-negative staphylococci from nostrils of healthy dogs and dog-owners in La Rioja. Spain Pathogens 13:229. 10.3390/pathogens13030229 38535572 10.3390/pathogens13030229 PMC10974962

[CR8] Abdullahi IN, Lozano C, Zarazaga M, Simón C, Höfle U, Sieber RN, Latorre-Fernández J, Stegger M, Torres C (2024c) Comparative genomics of *Staphylococcus aureus* strains from wild birds and pig farms elucidates levels of mobilomes, antibiotic pressure and host adaptation. J Global Antimicrobial Resistance 36:142–150. 10.1016/j.jgar.2023.12.003 10.1016/j.jgar.2023.12.003 38128728

[CR9] Aqel H, Sannan N, Foudah R (2023) From hospital to community: exploring antibiotic resistance and genes associated with virulence factor diversity of coagulase-positive *Staphylococci*. Antibiotics (basel, Switzerland) 12(7):1147. 10.3390/antibiotics12071147 37508243 10.3390/antibiotics12071147 PMC10376022

[CR10] Argemi X, Hansmann Y, Prola K, Prévost G (2019) Coagulase-negative staphylococci pathogenomics. Int J Mol Sci 20(5):1215. 10.3390/ijms20051215 30862021 10.3390/ijms20051215 PMC6429511

[CR11] Argudín MA, Butaye P (2016) Dissemination of metal resistance genes among animal methicillin-resistant coagulase-negative Staphylococci. Res Vet Sci 105:192–194. 10.1016/j.rvsc.2016.02.009 27033931 10.1016/j.rvsc.2016.02.009

[CR12] Arndt D, Grant JR, Marcu A, Sajed T, Pon A, Liang Y et al (2016) PHASTER: a better, faster version of the PHAST phage search tool. Nucleic Acids Res 44:W16–W21. 10.1093/nar/gkw387 27141966 10.1093/nar/gkw387 PMC4987931

[CR13] Bai B, Hu K, Zeng J, Yao W, Li D, Pu Z, Chen Z, Cheng H, Zheng J, Pan W, Lin Z, Xie L, Deng Q, Yu Z (2019) Linezolid consumption facilitates the development of linezolid resistance in Enterococcus faecalis in a tertiary-care hospital: a 5-year surveillance study. Microb Drug Resist 25(6):791–798 30762463 10.1089/mdr.2018.0005

[CR14] Banaszkiewicz S, Calland JK, Mourkas E, Sheppard SK, Pascoe B, Bania J (2019) Genetic diversity of composite enterotoxigenic Staphylococcus epidermidis pathogenicity islands. Genome Biol Evol 11(12):3498–3509. 10.1093/gbe/evz259 31769803 10.1093/gbe/evz259 PMC6931896

[CR15] Belhout C, Boyen F, Vereecke N, Theuns S, Taibi N, Stegger M, de la Fé-Rodríguez PY, Bouayad L, Elgroud R, Butaye P (2023) Prevalence and molecular characterization of methicillin-resistant staphylococci (MRS) and mammaliicocci (MRM) in dromedary camels from algeria: first detection of SCCmec-mecC hybrid in methicillin-resistant mammaliicoccus lentus. Antibiotics (Basel, Switzerland) 12(4):674. 10.3390/antibiotics12040674 37107036 10.3390/antibiotics12040674 PMC10134997

[CR16] Bortolaia V, Kaas RS, Ruppe E, Roberts MC, Schwarz S, Cattoir V, Philippon A, Allesoe RL, Rebelo AR, Florensa AF, Fagelhauer L, Chakraborty T, Neumann B, Werner G, Bender JK, Stingl K, Nguyen M, Coppens J, Xavier BB, Malhotra-Kumar S, Aarestrup FM (2020) ResFinder 4.0 for predictions of phenotypes from genotypes. J Antimicrobial Chemother 75(12):3491–3500. 10.1093/jac/dkaa345 10.1093/jac/dkaa345 PMC7662176 32780112

[CR17] Chajęcka-Wierzchowska W, Gajewska J, Zadernowska A, Randazzo CL, Caggia C (2023) A comprehensive study on antibiotic resistance among coagulase-negative staphylococci (CoNS) strains isolated from ready-to-eat food served in bars and restaurants. Foods (basel, Switzerland) 12(3):514. 10.3390/foods12030514 36766043 10.3390/foods12030514 PMC9914766

[CR18] Chang SC, Lin LC, Ge MC, Liu TP, Lu JJ (2019) Characterization of a novel, type II staphylococcal cassette chromosome mec element from an endemic oxacillin-resistant Staphylococcus lugdunensis clone in a hospital setting. J Antimicrob Chemother 74(8):2162–2165. 10.1093/jac/dkz189 31106369 10.1093/jac/dkz189

[CR19] Chang SC, Lin LC, Lu JJ (2021) Comparative genomic analyses reveal potential factors responsible for the ST6 oxacillin-resistant staphylococcus lugdunensis endemic in a hospital. Front Microbiol 12:765437. 10.3389/fmicb.2021.765437 10.3389/fmicb.2021.765437 PMC8655729 34899648

[CR20] Couvin D, Bernheim A, Toffano-Nioche C, Touchon M, Michalik J, Néron B, Rocha EPC, Vergnaud G, Gautheret D, Pourcel C (2018) CRISPRCasFinder, an update of CRISPRFinder, includes a portable version, enhanced performance and integrates search for Cas proteins. Nucleic Acids Res 46(W1):W246–W251. 10.1093/nar/gky425 29790974 10.1093/nar/gky425 PMC6030898

[CR21] Damavandi MS, Safarpour Dehkordi M, Dehghan A, Heibati F, Taghaddosi R et al (2017) Detection of antiseptic resistance genes among Staphylococcus aureus colonising nurses and coagulase-negative staphylococci isolated from clinical specimens at teaching hospitals in Southwest of Iran. Jundishapur J Microbiol 10(1):e39285. 10.5812/jjm.39285

[CR22] de Moura GS, de Carvalho E, Ramos Sanchez EM, Sellera FP, Marques MFS, Heinemann MB, De Vliegher S, Souza FN, Mota RA (2023) Emergence of livestock-associated Mammaliicoccus sciuri ST71 co-harbouring mecA and mecC genes in Brazil. Vet Microbiol 283:109792. 10.1016/j.vetmic.2023.109792 10.1016/j.vetmic.2023.109792 37269712

[CR23] Dimitriu T (2022) Evolution of horizontal transmission in antimicrobial resistance plasmids. Microbiology (Reading, England) 168(7). 10.1099/mic.0.001214 10.1099/mic.0.001214 35849537

[CR24] Dortet L, Glaser P, Kassis-Chikhani N, Girlich D, Ichai P, Boudon M, Samuel D, Creton E, Imanci D, Bonnin R, Fortineau N, Naas T (2018) Long-lasting successful dissemination of resistance to oxazolidinones in MDR Staphylococcus epidermidis clinical strains in a tertiary care hospital in France. J Antimicrob Chemother 73(1):41–51. 10.1093/jac/dkx370 29092052 10.1093/jac/dkx370 PMC5890688

[CR25] França A, Gaio V, Lopes N, Melo LDR (2021) Virulence factors in coagulase-negative staphylococci. Pathogens (basel, Switzerland) 10(2):170. 10.3390/pathogens10020170 33557202 10.3390/pathogens10020170 PMC7913919

[CR26] Gostev V, Leyn S, Kruglov A, Likholetova D, Kalinogorskaya O, Baykina M, Dmitrieva N, Grigorievskaya Z, Priputnevich T, Lyubasovskaya L, Gordeev A, Sidorenko S (2021) Global expansion of linezolid-resistant coagulase-negative staphylococci. Front Microbiol 12:661798. 10.3389/fmicb.2021.661798 34589061 10.3389/fmicb.2021.661798 PMC8473885

[CR27] Grazul M, Balcerczak E, Sienkiewicz M (2023) Analysis of the presence of the virulence and regulation genes from *Staphylococcus**aureus* (*S*. *aureus*) in coagulase negative staphylococci and the influence of the staphylococcal cross-talk on their functions. Int J Environ Res Public Health 20(6):5155. 10.3390/ijerph20065155 36982064 10.3390/ijerph20065155 PMC10049693

[CR28] Grispoldi L, Karama M, Armani A, Hadjicharalambous C, Cenci-Goga BT (2021) Staphylococcus aureus enterotoxin in food of animal origin and staphylococcal food poisoning risk assessment from farm to table. Ital J Anim Sci 20(1):677–690. 10.1080/1828051x.2020.1871428

[CR29] Harrison EM, Paterson GK, Holden MT, Ba X, Rolo J, Morgan FJ, Pichon B, Kearns A, Zadoks RN, Peacock SJ, Parkhill J, Holmes MA (2014) A novel hybrid SCCmec-mecC region in Staphylococcus sciuri. J Antimicrob Chemother 69(4):911–918. 10.1093/jac/dkt452 24302651 10.1093/jac/dkt452 PMC3956370

[CR30] Heilmann C, Ziebuhr W, Becker K (2019) Are coagulase-negative staphylococci virulent? Clin Microbiol Infection : off Pub Eur Soc Clin Microbiol Infectious Diseases 25(9):1071–1080. 10.1016/j.cmi.2018.11.012 10.1016/j.cmi.2018.11.012 30502487

[CR31] Huang L, Ahmed S, Gu Y, Huang J, An B, Wu C, Zhou Y, Cheng G (2021) The effects of natural products and environmental conditions on antimicrobial resistance. Molecules (Basel, Switzerland) 26(14):4277. 10.3390/molecules26144277 34299552 10.3390/molecules26144277 PMC8303546

[CR32] Idrees M, Sawant S, Karodia N, Rahman A (2021) *Staphylococcus aureus* biofilm: morphology, genetics, pathogenesis and treatment strategies. Int J Environ Res Public Health 18(14):7602. 10.3390/ijerph18147602 34300053 10.3390/ijerph18147602 PMC8304105

[CR33] Jolley KA, Bray JE, Maiden MCJ (2018) Open-access bacterial population genomics: BIGSdb software, the PubMLST.org website and their applications. Wellcome Open Res 3:124. 10.12688/wellcomeopenres.14826.1 30345391 10.12688/wellcomeopenres.14826.1 PMC6192448

[CR34] Kalai S, Roychoudhury P, Dutta TK, Subudhi PK, Chakraborty S, Barman NN, Sen A (2021) Multidrug resistant staphylococci isolated from pigs with exudative epidermitis in North eastern Region of India. Lett Appl Microbiol 72(5):535–541. 10.1111/lam.13448 33421175 10.1111/lam.13448

[CR35] Kirk F, Mashicharan M, Braddick M, Saxena P (2022) *Staphylococcus hyicus*, a novel pathogen causing destructive infective endocarditis requiring mitral annular reconstruction. JTCVS Techniques 13:70–73. 10.1016/j.xjtc.2022.03.008 35711206 10.1016/j.xjtc.2022.03.008 PMC9196931

[CR36] Kranjec C, Kristensen SS, Bartkiewicz KT, Brønner M, Cavanagh JP, Srikantam A, Mathiesen G, Diep DB (2021) A bacteriocin-based treatment option for Staphylococcus haemolyticus biofilms. Sci Rep 11(1):13909. 10.1038/s41598-021-93158-z 34230527 10.1038/s41598-021-93158-z PMC8260761

[CR37] Lawal OU, Fraqueza MJ, Bouchami O, Worning P, Bartels MD, Gonçalves ML, Paixão P, Gonçalves E, Toscano C, Empel J, Urbaś M, Domínguez MA, Westh H, de Lencastre H, Miragaia M (2021a) Foodborne origin and local and global spread of *Staphylococcus saprophyticus* causing human urinary tract infections. Emerg Infect Dis 27(3):880–893 33622483 10.3201/eid2703.200852 PMC7920669

[CR38] Lawal OU, Fraqueza MJ, Worning P, Bouchami O, Bartels MD, Goncalves L, Paixão P, Goncalves E, Toscano C, Empel J, Urbaś M, Domínguez MA, Westh H, de Lencastre H, Miragaia M (2021b) *Staphylococcus saprophyticus* causing infections in humans is associated with high resistance to heavy metals. Antimicrob Agents Chemother 65(7):e0268520. 10.1128/AAC.02685-20 33941519 10.1128/AAC.02685-20 PMC8218656

[CR39] Letunic I, Bork P (2021) Interactive Tree Of Life (iTOL) v5: an online tool for phylogenetic tree display and annotation. Nucleic Acids Res 49(W1):W293–W296. 10.1093/nar/gkab301 33885785 10.1093/nar/gkab301 PMC8265157

[CR40] Li Z, Peres AG, Damian AC, Madrenas J (2015) Immunomodulation and disease tolerance to Staphylococcus aureus. Pathogens (basel, Switzerland) 4(4):793–815. 10.3390/pathogens4040793 26580658 10.3390/pathogens4040793 PMC4693165

[CR41] Li Y, Gou H, Chu P, Zhang K, Jiang Z, Cai R, Song S, Bian Z, Li C (2021) Comparison of host cytokine response in piglets infected with toxigenic and non-toxigenic *Staphylococcus hyicus*. Front Veterinary Sci 8:639141. 10.3389/fvets.2021.639141 10.3389/fvets.2021.639141 PMC7920954 33665221

[CR42] Lin S, Sun B, Shi X, Xu Y, Gu Y, Gu X, Ma X, Wan T, Xu J, Su J, Lou Y, Zheng M (2021) Comparative genomic and pan-genomic characterization of *Staphylococcus epidermidis* From Different Sources Unveils the Molecular Basis and Potential Biomarkers of Pathogenic Strains. Front Microbiol 12:770191. 10.3389/fmicb.2021.770191 34867904 10.3389/fmicb.2021.770191 PMC8634615

[CR43] Loncaric I, Kübber-Heiss A, Posautz A, Ruppitsch W, Lepuschitz S, Schauer B, Feßler AT, Krametter-Frötscher R, Harrison EM, Holmes MA, Künzel F, Szostak MP, Hauschild T, Desvars-Larrive A, Misic D, Rosengarten R, Walzer C, Slickers P, Monecke S, Ehricht R, Spergser J (2019) Characterization of mecC gene-carrying coagulase-negative Staphylococcus spp. isolated from various animals. Veterinary Microbiol 230:138–144. 10.1016/j.vetmic.2019.02.014 10.1016/j.vetmic.2019.02.014 30827379

[CR44] Makarova KS, Wolf YI, Alkhnbashi OS, Costa F, Shah SA, Saunders SJ, Barrangou R, Brouns SJ, Charpentier E, Haft DH, Horvath P, Moineau S, Mojica FJ, Terns RM, Terns MP, White MF, Yakunin AF, Garrett RA, van der Oost J, Backofen R, Koonin EV (2015) An updated evolutionary classification of CRISPR-Cas systems. Nat Rev Microbiol 13(11):722–736. 10.1038/nrmicro3569 26411297 10.1038/nrmicro3569 PMC5426118

[CR45] Matuszewska M, Murray GGR, Ba X, Wood R, Holmes MA, Weinert LA (2022) Stable antibiotic resistance and rapid human adaptation in livestock-associated MRSA. Elife 11:e74819. 10.7554/eLife.74819 35762208 10.7554/eLife.74819 PMC9239682

[CR46] Merrild E, Winther M, Dahl JN, Ebsen TS, Leth S, Winther S (2023) Case report: rare case of *Staphylococcus pasteuri* endocarditis. Case Rep Cardiol 2023:4624492. 10.1155/2023/4624492 37013024 10.1155/2023/4624492 PMC10066806

[CR47] Murugesan AC, Varughese HS (2022) Analysis of CRISPR–Cas system and antimicrobial resistance in Staphylococcus coagulans strains. Lett Appl Microbiol 75(1):126–134. 10.1111/lam.13713 35366350 10.1111/lam.13713

[CR48] Nanoukon C, Affolabi D, Keller D, Tollo R, Riegel P, Baba-Moussa L, Prévost G (2018) Characterization of human type c enterotoxin produced by Clinical S. epidermidis Strains. Toxins 10(4):139. 10.3390/toxins10040139 29584685 10.3390/toxins10040139 PMC5923305

[CR49] Nasaj M, Saeidi Z, Tahmasebi H, Dehbashi S, Arabestani MR (2020) Prevalence and distribution of resistance and enterotoxins/enterotoxin-like genes in different clinical strains of coagulase-negative Staphylococcus. Eur J Med Res 25(1):48. 10.1186/s40001-020-00447-w 33046122 10.1186/s40001-020-00447-w PMC7552519

[CR50] Naushad, S., Naqvi, S. A., Nobrega, D., Luby, C., Kastelic, J. P., Barkema, H. W., & De Buck, J. (2019). Comprehensive virulence gene profiling of bovine non-aureus staphylococci based on whole-genome sequencing data. mSystems, 4(2), e00098-18. https://doi.org/10.1128/mSystems.00098-18 10.1128/mSystems.00098-18 PMC6401416 30863792

[CR51] Nishimasu H, Nureki O (2017) Structures and mechanisms of CRISPR RNA-guided effector nucleases. Curr Opin Struct Biol 43:68–78. 10.1016/j.sbi.2016.11.013 27912110 10.1016/j.sbi.2016.11.013

[CR52] Pal C, Bengtsson-Palme J, Rensing C, Kristiansson E, Larsson DG (2014) BacMet: antibacterial biocide and metal resistance genes database. Nucleic Acids Res 42(D1):D737–D743. 10.1093/nar/gkt1252 24304895 10.1093/nar/gkt1252 PMC3965030

[CR53] Paterson GK (2020) Genomic epidemiology of methicillin-resistant Staphylococcus sciuri carrying a SCCmec-mecC hybrid element. Infection, Genetics and Evolution : Journal of Molecular Epidemiology and Evolutionary Genetics in Infectious Diseases 79:104148. 10.1016/j.meegid.2019.104148 10.1016/j.meegid.2019.104148 31862259

[CR54] Podkowik M, Seo KS, Schubert J, Tolo I, Robinson DA, Bania J, Bystroń J (2016) Genotype and enterotoxigenicity of Staphylococcus epidermidis strain from ready to eat meat products. Int J Food Microbiol 229:52–59. 10.1016/j.ijfoodmicro.2016.04.013 27105039 10.1016/j.ijfoodmicro.2016.04.013 PMC4877272

[CR55] Rebelo A, Mourão J, Freitas AR, Duarte B, Silveira E, Sanchez-Valenzuela A, Almeida A, Baquero F, Coque TM, Peixe L, Antunes P, Novais C, & from the ESCMID Study Group on Food- and Water-borne Infections (EFWISG) (2021) Diversity of metal and antibiotic resistance genes in Enterococcus spp. from the last century reflects multiple pollution and genetic exchange among phyla from overlapping ecosystems. Sci Total Environ 787:147548. 10.1016/j.scitotenv.2021.147548 10.1016/j.scitotenv.2021.147548 34000557

[CR56] Rossi CC, Souza-Silva T, Araújo-Alves AV, Giambiagi-deMarval M (2017) CRISPR-Cas systems features and the gene-reservoir role of coagulase-negative staphylococci. Front Microbiol 8:1545. 10.3389/fmicb.2017.01545 28861060 10.3389/fmicb.2017.01545 PMC5559504

[CR57] Rossi CC, Pereira MF, Giambiagi-deMarval M (2020) Underrated Staphylococcus species and their role in antimicrobial resistance spreading. Genet Mol Biol 43(1 suppl 2):e20190065. 10.1590/1678-4685-GMB-2019-0065 32052827 10.1590/1678-4685-GMB-2019-0065 PMC7198029

[CR58] Ruiz-Ripa L, Feßler AT, Hanke D, Eichhorn I, Azcona-Gutiérrez JM, Alonso CA, Pérez-Moreno MO, Aspiroz C, Bellés A, Schwarz S, Torres C (2021) Mechanisms of linezolid resistance among clinical Staphylococcus spp. in Spain: spread of methicillin- and linezolid-resistant Epidermidis ST2. Microb Drug Resist 27(2):145–153 32456543 10.1089/mdr.2020.0122

[CR59] Saber H, Jasni AS, Jamaluddin TZMT, Ibrahim R (2017) A review of staphylococcal cassette chromosome *mec* (SCC*mec*) types in coagulase-negative staphylococci (CoNS) species. Malaysian J Med Sci : MJMS 24(5):7–18 10.21315/mjms2017.24.5.2 PMC5772811 29386968

[CR60] Sahl JW, Lemmer D, Travis J, Schupp JM, Gillece JD, Aziz M, Driebe EM, Drees KP, Hicks ND, Williamson CHD, Hepp CM, Smith DE, Roe C, Engelthaler DM, Wagner DM, Keim P (2016) NASP: an accurate, rapid method for the identification of SNPs in WGS datasets that supports flexible input and output formats. Microbial Genomics 2(8):e000074. 10.1099/mgen.0.000074 28348869 10.1099/mgen.0.000074 PMC5320593

[CR61] Santoiemma PP, Kalainov DM, Mehta MP, Bolon MK (2020) An unusual case of *Staphylococcus pasteuri* osteomyelitis. Infect Dis Rep 12(2):8523. 10.4081/idr.2020.8523 32913620 10.4081/idr.2020.8523 PMC7459742

[CR62] Schijffelen MJ, Boel CH, van Strijp JA, Fluit AC (2010) Whole genome analysis of a livestock-associated methicillin-resistant Staphylococcus aureus ST398 isolate from a case of human endocarditis. BMC Genomics 11:376. 10.1186/1471-2164-11-376 20546576 10.1186/1471-2164-11-376 PMC2900268

[CR63] Seier-Petersen MA, Nielsen LN, Ingmer H, Aarestrup FM, Agersø Y (2015) Biocide Susceptibility of Staphylococcus aureus CC398 and CC30 Isolates from Pigs and Identification of the Biocide Resistance Genes, qacG and qacC. Microb Drug Resist (Larchmont, N.Y.) 21(5):527–536. 10.1089/mdr.2014.0215 10.1089/mdr.2014.0215 26430941

[CR64] Shmakov S, Abudayyeh OO, Makarova KS, Wolf YI, Gootenberg JS, Semenova E, Minakhin L, Joung J, Konermann S, Severinov K, Zhang F, Koonin EV (2015) Discovery and functional characterization of diverse class 2 CRISPR-Cas systems. Mol Cell 60(3):385–397. 10.1016/j.molcel.2015.10.008 26593719 10.1016/j.molcel.2015.10.008 PMC4660269

[CR65] Shokrollahi P, Hasani A, Aghazadeh M, Memar MY, Hasani A, Zaree M, Rezaee MA, Sadeghi J (2022) Contribution of arginine catabolic mobile element and copper and mercury resistance element in methicillin-resistant *Staphylococcus**aureus*: a vantage point. Canadian J Infect Dis Med Microbiol 2022(1):9916255 10.1155/2022/9916255 PMC9637032 36345550

[CR66] Slifierz MJ, Friendship RM, Weese JS (2015) Methicillin-resistant Staphylococcus aureus in commercial swine herds is associated with disinfectant and zinc usage. Appl Environ Microbiol 81(8):2690–2695. 10.1128/AEM.00036-15 25662976 10.1128/AEM.00036-15 PMC4375337

[CR67] Souza-Silva T, Rossi CC, Andrade-Oliveira AL, Vilar LC, Pereira MF, Penna BA, Giambiagi-deMarval M (2022) Interspecies transfer of plasmid-borne gentamicin resistance between *Staphylococcus* isolated from domestic dogs to Staphylococcus aureus. Infect, Genetics Evolution : J Mol Epidemiol Evolutionary Genetics Infect Dis 98:105230. 10.1016/j.meegid.2022.105230 10.1016/j.meegid.2022.105230 35104683

[CR68] Stach CS, Vu BG, Schlievert PM (2015) Determining the presence of superantigens in coagulase negative staphylococci from humans. PLoS ONE 10(11):e0143341. 10.1371/journal.pone.0143341 26599862 10.1371/journal.pone.0143341 PMC4658126

[CR69] Szczuka E, Wesołowska M, Krawiec A, Kosicki JZ (2023) Staphylococcal species composition in the skin microbiota of domestic pigeons (Columba livia domestica). PLoS ONE 18(7):e0287261. 10.1371/journal.pone.0287261 37436966 10.1371/journal.pone.0287261 PMC10337865

[CR70] Tao S, Chen H, Li N, Liang W (2022a) The application of the CRISPR-Cas system in antibiotic resistance. Infect Drug Resistance 15:4155–4168. 10.2147/IDR.S370869 10.2147/IDR.S370869 PMC9356603 35942309

[CR71] Veras JF, do Carmo LS, Tong LC, Shupp JW, Cummings C, Dos Santos DA, Jett M (2008) A study of the enterotoxigenicity of coagulase-negative and coagulase-positive staphylococcal isolates from food poisoning outbreaks in Minas Gerais Brazil. Int J Infect Dis 12(4):410–415 18206412 10.1016/j.ijid.2007.09.018

[CR72] Zankari E, Allesøe R, Joensen KG, Cavaco LM, Lund O, Aarestrup FM (2017) PointFinder: a novel web tool for WGS-based detection of antimicrobial resistance associated with chromosomal point mutations in bacterial pathogens. J Antimicrob Chemother 72(10):2764–2768. 10.1093/jac/dkx217 29091202 10.1093/jac/dkx217 PMC5890747

