# Supplementary material for: Genomic analysis of multi-drug resistant coagulase-negative staphylococci from healthy humans and animals revealed unusual mechanisms of resistance and CRISPR-Cas system
Source: Int Microbiol. 2024 Sep 17;28(5):941–63. doi: 10.1007/s10123-024-00577-9 (PMC12162761; doi:10.1007/s10123-024-00577-9)
Supplement: Supplementary file 1 — Supplementary file1 (DOCX 21 KB) [file 10123_2024_577_MOESM1_ESM.docx]

**Supplementary Table S1.** Comparison of the level of MDR among CoNS isolates from the different hosts^a^: healthy humans with or without contact with animals and healthy animals (nestling storks, pigs and dogs) and characteristics of the 26 MDR CoNS isolates analyzed in this study

| ^b^Non-duplicated strains | Nestling storks (n=268) | Healthy Pigs (n =62) | Healthy Pig farmers (n=13) | Healthy dogs (n=32) | Healthy Dog owners (n=98) | Healthy humans (no animal contact) (n =116) |
| --- | --- | --- | --- | --- | --- | --- |
| MDR phenotype (≥4 classes of antibiotics)  Yes  No | 13  255 | 52  10 | 13  0 | 9  23 | 32  66 | 10  106 |
| ^c^Strains analysed by WGS | **Nestling storks (n=4)** | **Healthy Pigs (n =14)** | **Healthy Pig farmers (n=)** | **Healthy Dogs (n =1)** | **Healthy Dog owners (n=3)** | **Healthy humans (no animal contact) (n =4)** |
| AMR phenotypes ^(number of strains)^ | 1. *S. haemolyticus* (PEN, FOX, CLI, TET, SXT, GEN, TOB) 2. *S. epidermidis* (PEN, ERY, FOS, MUP ) 3. *S. lentus* (PEN, FOX, ERY, TET, FOS) 4. *S. arlette* (PEN, ERY, CLI, TET, GEN, CIP) | 1. *S. borealis* (PEN^4^, FOX^4^, ERY^4^, CLI^4^, TET^4^, SXT^4^, TOB^3^, STR^4^, AMK^2^, BLE^4^, SPE^3^, CLO^2^, CIP^4^) 2. *S. saprophyticus* (PEN^1^, FOX^2^, ERY^2^, CLI^2^, TET^2^, SXT^2^, GEN^1^, TOB^2^, STR^2^, AMK^1^, FUS^1^, CLO^1^, **LZD**^1^) 3. *S. sciuri* (PEN, FOX, ERY, CLI, TET, SXT, GEN, TOB, SPE, STR, BLE, CLO) 4. *S. pastueri* (PEN, FOX, ERY, CLI, TET, SXT, TOB, STR) 5. *S. hyicus* (PEN^2^, ERY^2^, CLI^2^, TET^2^, SXT^2^, GEN^1^, TOB^1^, STR^2^) 6. *S. simulans* PEN, ERY, CLI, SPE, CIP) | 1. *S. saprophyticus* (PEN, FOX, ERY, CLI, TET, SXT, GEN, TOB, STR, AMK, FUS)  2. *S. haemolyticus* (PEN, FOX, CLI, TET, SXT, GEN)  3. *S. epidermidis* (PEN, FOX, CLI, TET, SXT, TOB, STR, CLO, **LZD**, FOS) | 1. *S. epidermidis* (PEN^3^, FOX^2^, ERY^3^, CLI^2^, TET^2^, SXT^1^, TOB^1^, BLE^1^, SPE^1^, **LZD^1^,** FOS^3^, FUS^1^, MUP^2^) | 1. *S. hominis* (PEN, FOX, ERY, TET, TOB, SXT, BLE, FUS) | 1. *S. epidermidis* (PEN^3^, FOX^1^, ERY^2^, CLI^3^, TET^2^, SXT^2^, GEN^1^, TOB^2^, FOS^3^, MUP^1^, CIP^2^)  2. *S. haemolyticus* (PEN, FOX, ERY, TET, SXT, GEN, TOB, CIP) |

^a^The total number of CoNS isolates included in this table to determine the rate of MDR in the different hosts was obtained from data previously published (Abdullahi *et al*., 2023a; 2023b; 2023c; 2024a).

^b^Non-duplicated strains are those of different individuals or different species or different AMR phenotypes

^c^These included (i) CoNS that presented the MDR phenotype for four or more classes of antibiotics and (ii) one species each per host carrying the MDR (iii) MDR-CoNS with similar AMR genes detected from humans and animals in the same ecological niche.

AMK: Amikacin; BLE: Bleomycin, CHL: Chloramphenicol; CLI: clindamycin; CIP: ciprofloxacin; ERY: erythromycin; FOS: Fosfomycin; FOX: cefoxitin; FUS: Fusidic acid; GEN: gentamicin; LZD: linezolid; MUP: mupirocin; PEN: penicillin; SPE: Spectinomycin; SXT: sulfamethoxazole/trimethoprim; STR: Streptomycin; TET: tetracycline, TOB: tobramycin.
